# Toxicological Exploration of Collagenase and Benzonase on Rat Precision-Cut Liver Slices

**DOI:** 10.1021/acs.jproteome.5c01232

**Published:** 2026-09-17

**Authors:** Janine Stam, Peter Olinga, Aswin Menke, Pieter Annaert, Klaas Nico Faber, Peter L. Horvatovich, Rainer Bischoff, Justina C. Wolters

**Affiliations:** † Department of Analytical Biochemistry, Groningen Research Institute of Pharmacy, 3647 University of Groningen, 9700 AD Groningen, The Netherlands; ‡ Department of Pharmaceutical Technology and Biopharmacy, Groningen Research Institute of Pharmacy, University of Groningen, 9713 AV Groningen, The Netherlands; § Department of Metabolic Health Research, The Netherlands Organization for Applied Scientific Research (TNO), Leiden 2333 BE, The Netherlands; ∥ Drug Delivery and Disposition, Department of Pharmaceutical and Pharmacological Sciences, KU Leuven, Leuven B-3000, Belgium; ⊥ BioNotus CommV, Niel 2845, Belgium; # Department of Gastroenterology and Hepatology, University of Groningen, University Medical Center Groningen, 9713 GZ Groningen, The Netherlands; ∇ Department of Pediatrics, University of Groningen, University Medical Center Groningen, 9713 AV Groningen, The Netherlands

**Keywords:** precision-cut liver slices, extracellular vesicles, collagenase, benzonase, proteomics

## Abstract

Precision-cut liver slices (PCLSs) are an *ex vivo* liver model that allows isolation of extracellular vesicles (EVs), which are nano-sized particles involved in intercellular communication. However, EV recovery from PCLSs is hindered by adhesion to the extracellular matrix and macromolecules such as extracellular DNA. Enzymatic treatments, such as collagenase (COL) and the endonuclease benzonase (BEN), can aid their release, but potential effects on the tissue are unclear. Here, the effect of these enzymes separately, as well as combined, was analyzed to determine their effects on morphology, protein content, and the proteome of the PCLS. The enzymes showed minimal effects on cell viability and protein content after two different incubation times, whereas morphological analysis confirmed collagen degradation by COL and COL­(BEN), along with decreased ribosomal and secretory secretory protein levels. BEN decreased the levels of proteins related to protein synthesis and degradation. Increased levels of proteins related to detoxification and energy production were observed upon addition of COL, BEN, and COL­(BEN). This study emphasizes the potential and risks for the use of collagenase and benzonase for EV isolation from the PCLS.

## Graphical abstract

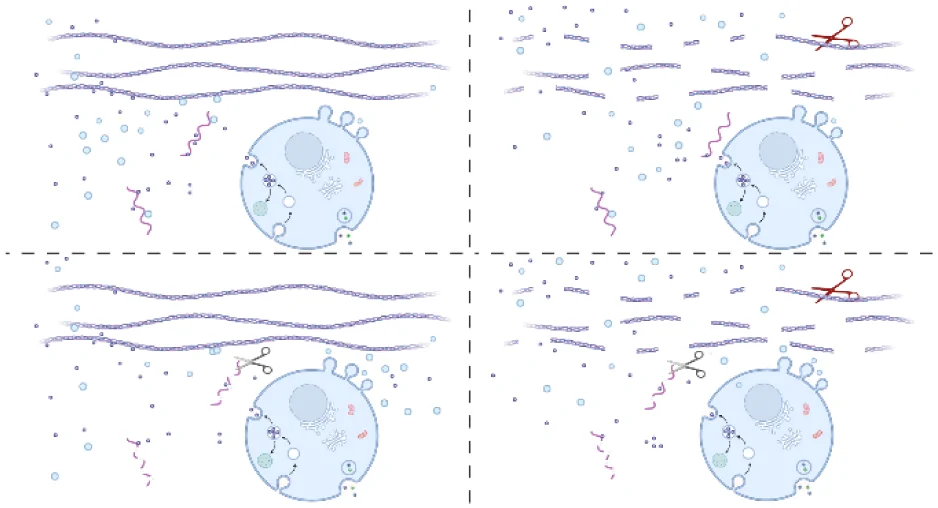

## Introduction

The liver is a vital organ responsible for numerous essential physiological functions, including detoxification, metabolism, and bile production. Despite its regenerative capacity, the rising global burden of liver diseases highlights its vulnerability to various lifestyle and nonlifestyle stresses. As a result, hepatic conditions such as drug-induced liver injury (DILI), viral hepatitis, alcohol-associated liver disease (ALD), primary sclerosing cholangitis (PSC), liver cirrhosis, hepatocellular carcinoma (HCC), and metabolic dysfunction-associated steatotic liver disease (MASLD) are increasingly prevalent.^1^

To study liver diseases, researchers leverage a variety of experimental models, ranging from *in vivo* systems that preserve physiological complexity to *in vitro* models that allow for detailed mechanistic studies. *Ex vivo* models, such as precision-cut liver slices (PCLS), have emerged as a promising tool in hepatic research.^2–4^ These models bridge the gap between *in vivo* and *in vitro* approaches by maintaining tissue architecture, cell–cell interactions, and metabolic activity using human tissue while limiting the number of animals used per experiment.^5^ PCLSs have proven to be particularly valuable in the study of drug metabolism, liver toxicity, fibrosis, and metabolic liver diseases such as MASLD and cholestatic liver disorders, where dynamic interactions between multiple cell types are involved in these complex processes.^3,4,6–9^ These *ex vivo* models provide an additional benefit when studying extracellular products since, unlike *in vivo* systems, they provide a controlled environment to analyze only extracellular products such as extracellular vesicles (EVs) derived directly from the tissue under investigation.

Extracellular vesicles are nanosized, membrane-enclosed particles secreted by virtually all cell types, and they play a pivotal role in intercellular communication.^10^ EVs reflect their cell of origin’s state at the time of release, making them valuable targets for identification of diagnostic and prognostic biomarkers and the potential identification of therapeutic targets.^11,12^ However, one significant challenge in the use of *ex vivo* models is the isolation of EV from the extracellular microenvironment due to factors such as adhesion to the extracellular matrix (ECM) in the absence of shear stress due to no blood flow or interactions with macromolecules like extracellular DNA.^13^ To address these challenges, enzymatic treatments with collagenase and DNAse have been used, though their potential effects on tissue integrity and the composition of the proteome remain poorly understood.^11,13–22^

In this study, we employ the PCLS model to investigate the impact of treatments with collagenase and benzonase (a DNA/RNA nonspecific nuclease)^23^ on PCLS viability and PCLS morphology as well as on the composition of the PCLS proteome. This study aims to identify which factors in the PCLS are affected by the methodological approaches aimed at improving the EV isolations from *ex vivo* liver models, ultimately enhancing their utility in hepatic disease research. By providing a comprehensive evaluation of collagenase and benzonase treatments, this study helps guide the use of these enzymes in EV research using PCLS. This is particularly relevant for liver-specific diseases, as PCLSs offer a unique platform to investigate dynamic, tissue-specific processes while preserving the complexity of the liver microenvironment.

## Materials and Methods

### Animals Used

One female and five male Wistar rats (250–350 g) were purchased from Charles River (Sulzfeld, Germany) and were allowed to adapt to the new environment for at least 7 days before the experiment started. Housing took place in a temperature- and humidity-controlled room on a 12-h light/dark cycle (Central Animal Facility, University Medical Center Groningen, The Netherlands) with water and the Teklad-irradiated 2918 diet (Envigo, Horst, The Netherlands) continuously available. All experiments started in the morning (between 9:00 and 10:00 am) to avoid experimental variability due to circadian rhythms. The livers were extracted from the rats under general isoflurane/O_2_ anesthesia (Pharmachemie B.V., member of the TEVA Group, Haarlem, the Netherlands) with the use of an anesthetic machine (Datex Ohmeda Tec 5 Isoflurane Vaporizer, Rob Vollmer te Uithuizen (RVU) (Uithuizen, The Netherlands) and were immediately stored in ice-cold University of Wisconsin preservation solution (UW, DuPont Critical Care, Waukegan, IL, USA). The Animal Ethical Committee of the University of Groningen approved all experiments (CCD-license: 105002017884), and experiments were performed in accordance with the EU directive 2010/63/EU for animal experiments.

### Preparation of Rat Precision-Cut Liver Slices

After liver extraction, rat precision-cut liver slices (PCLS) were prepared as described before.^4^ In brief, cylindrical cores were manually cut from the tissue with 6 mm biopsy punches (Kai Medical, Solingen, Germany). Each core was sliced into small round slices with a thickness of ± 250 μm using a Krumdieck tissue slicer (Alabama Research and Development, Munford, USA) that was filled with ice-cold Krebs-Henseleit buffer (KHB) supplemented with 25 mM d-glucose (Merck, Schiphol-Rijk, The Netherlands), 25 mM NaHCO_3_ (Merck, Schiphol-Rijk, The Netherlands), and 10 mM 4-(2-hydroxyethyl)-1-piperazineethanesulfonic acid (HEPES) (MP Biomedicals, Aurora, USA) that was fumigated in advance with carbogen (95% O_2_ + 5% CO_2_) and adjusted to a pH of 7.4. Immediately after slicing, PCLS were transferred to UW to avoid a loss in viability. The wet weight of each slice was ±5 mg.

### Incubation of Rat Precision-Cut Liver Slices

Incubation of PCLS was done in 12-well plates filled with 1.3 mL pre-warmed and pre-saturated (80% O_2_ + 5% CO_2_) William’s Medium E + GlutaMAX (Thermo Scientific, Groningen, The Netherlands) (WME) supplemented with 25 mM d-glucose (Merck, Schiphol-Rijk, The Netherlands) and 50 μg/mL gentamycin (Thermo Scientific, Groningen, The Netherlands) per well. Before starting, all slices were pre-incubated for 1 h to allow them to recover and to remove any cell debris resulting from the slicing process. Afterward, slices were transferred to new plates. Depending on the experiment, slices were either incubated for 24 or 48 h with or without collagenase D (Merck, Schiphol-Rijk, The Netherlands) (COL), benzonase nuclease (Merck, Schiphol-Rijk, The Netherlands) (BEN), or both enzymes together (COLBEN). Details regarding the origin, batch differences of collagenase, and possible contaminations are listed in Supporting File 1, Table S1. For the analysis of ATP content, LDH leakage, and total protein content, rat PCLS were incubated with COL concentrations of 0, 1, 1.5, 2, 3, or 4 mg/mL and BEN concentrations of 0, 25, 50, 62.5, 75, or 100 U/mL. For the combination of both enzymes (COLBEN), a fixed concentration of 50 U/mL BEN was chosen and variable concentrations of COL (0, 1, 1.5, 2, 3, or 4 mg/mL). In the case of a 48 h incubation, the first 24 h were without COL, BEN, or COLBEN. Slices were transferred to new incubation plates after 24 h.

### ATP Content Determination

In brief, after incubation, each slice was transferred to a tube filled with 1 mL sonification solution (70% v/v ethanol, 2 mM ethylenediaminetetraacetic acid, pH 10.9) that was subsequently snap-frozen in liquid nitrogen. After that, all samples were stored at −80 °C. On the analysis day, samples were homogenized with a Mini-Beadbeater-8 (BioSpec Products, Bartlesville, USA) for 45 s and subsequently centrifuged at 13,200 rpm (Eppendorf Centrifuge 5424 R). The supernatant was kept on ice and used for ATP content analysis, while the pellet was dried at 37 °C for later protein content analysis.^4^

For ATP content analysis, the ATP Bioluminescence Assay kit CLS II (Roche, Mannheim, Germany) was used according to the manufacturer’s protocol with minor modifications. In brief, samples were diluted 10 times with 0.1 M Tris-HCl containing 2 mM EDTA (pH 7.8). Using a standard ATP calibration curve, luminescence was measured with a Synergy HT plate reader (BioTek, Winooski, USA) in black 96-well plates.

### Protein Content Determination

The protein assay was performed based on the protein assay sample preparation method as described by Starokozhko et al.,^24^ followed by the Bio-Rad DC Protein Assay (Bio-Rad, Munich, Germany) with bovine serum albumin for calibration. The absorbance was measured on a Synergy HT plate reader (BioTek, Winooski, USA) at 650 nm after keeping the plate in the dark for 15 min.

### LDH Leakage Assessment

Lactate dehydrogenase (LDH) leakage was measured to determine the degree of cell membrane damage, as described previously.^25^ The CytoTox-ONE Homogenous Membrane Integrity Assay kit (Promega, Madison, USA) was used according to the manufacturer’s protocol with minor modifications. The conditioned medium per slice was collected after 24 and 48 h of incubation and stored at 4 °C for a maximum of 1 week. Control slices per experiment were collected after the 1-h pre-incubation period and stored at −80 °C. All collected material was thawed to room temperature on the day of analysis. The control slices were homogenized in 1.3 mL WME with a Mini-BeadBeater-8 (BioSpec Products, Bartlesville, USA) and subsequently centrifuged for 2 min at 13,200 rpm (Eppendorf centrifuge 5424 R). Samples were mixed with substrate mix in a 1:1 ratio in a black 96-well plate. After incubation for 10.5 min in the dark (plate covered with aluminum foil), stop solution was added to all samples, and fluorescence counts were immediately measured at an excitation of 560 nm and an emission of 590 nm after 5 seconds of automatic mixing on a SpectraMax Gemini, XPS (Molecular Devices, San Jose, USA). The LDH leakage of samples that were incubated for 24 h (LDH%_s24_) and 48 h (LDH%_s48_) was calculated as follows:

1 $$LDH{\%}_{s24}=\frac{\left(FC_{s24}-FC_{bl}\right)}{\left(FC_{Ctrl}-FC_{bl}\right)}\cdot 100$$

2 $$LDH{\%}_{s48}=\frac{\left(FC_{s24}-FC_{bl}\right)+\left(FC_{s48}-FC_{bl}\right)}{\left(FC_{Ctrl}-FC_{bl}\right)}\cdot 100$$

where FC stands for the fluorescence counts of sample with conditioned media between 0–24 h (s24) or 24–48 h (s48) of incubation time, blank culture medium (bl), or homogenized control slices (ctrl).

### Morphological Analyses

Preparation of PCLS for morphological examination was performed as described previously with some modifications.^26^ In brief, 3 PCLS for every condition per rat (at least 3 rats for every condition) were fixated in 4% formalin for 24 h at 4 °C and subsequently stored in 70% aq. ethanol at 4 °C until further analysis. Next, slices were dehydrated in ethanol baths with increasing concentrations, followed by xylene and paraffine baths and with the aid of an Excelsior AS tissue processor set on the routine night protocol (Thermo Scientific, Groningen, The Netherlands). Afterward, they were embedded at 62 °C in paraffin aligned parallel to the slice surface (i.e., the flat part of the slice had the same direction as the flat part of the paraffin) according to standard procedure. After cooling, 4 μm sections were cut from the embedded slices with a Reichert Jung 2040 Biocut Microtome (Cambridge Instruments GmbH, UK), followed by deparaffinization and rehydration. Sections were either stained using hematoxylin and eosin (H&E) (Merck, Schiphol-Rijk, The Netherlands) to study the overall morphology or using a Sirius Red staining solution according to the manufacturer’s protocol (Abcam, Cambridge, UK) to study the collagen-containing extracellular matrix. All sections were scanned with a Pannoramic P250 Scanner (3DHistech, Budapest, Hungary). To assess single-cell necrosis, a pathologist manually counted the number of necrotic cells in five randomly selected areas of each tissue section.

### Proteomics Sample Preparation

After 24 and 48 h of incubation, relative levels of individual proteins present in the PCLS were determined with label-free proteomics. At the end of the incubation period, slices were placed in empty tubes, snap-frozen in liquid nitrogen, and subsequently stored at −80 °C. On the day of analysis, slices were thawed to room temperature and homogenized in 150 μL of NP40 lysis solution (0.1% IGEPAL CA-630, 0.4 M NaCl, 10 mM Tris-HCl (pH 8.0), 1 mM EDTA) using a Mini-BeadBeater 24 (Biospec Products, Bartlesville, USA). Subsequently, in-gel trypsin digestion and further sample cleanup were performed as previously described with some modifications.^27^ In brief, 15 μg protein per sample (24 h samples) or 10.5 μg protein per sample (48 h samples) as determined by a Bradford assay beforehand (Bio-Rad, Hercules, California, USA) was loaded in a 3:1 ratio with 4× LDS sample buffer (Abcam, Cambridge, UK) on a pre-cast 4–12% Bis-Tris gel (Abcam, Cambridge, UK). The gels ran for ±5 min at 100 V, were stained with InstantBlue Coomassie Protein stain (Abcam, Cambridge, UK), and washed with ultrapure water. One band per sample containing all proteins was excised from the gel and cut into 6–8 smaller pieces. Staining was removed by repeated washes with 30% acetonitrile (ACN)/70% 100 mM NH_4_HCO_3_ (ABC) and dehydration was performed in 50% ACN/50% 100 mM ABC for 30 min, followed by 100% ACN for 5 min. After drying the gel pieces in an oven at 37 °C, reduction was performed in 10 mM DL-Dithiothreitol (Sigma-Aldrich) followed by alkylation in 55 mM iodoacetamide (Sigma-Aldrich), both for 30 min, which was dissolved in 100 mM ABC. Trypsin digestion was performed overnight at 37 °C in a 1:100 w/w ratio of sequencing grade modified trypsin (Promega) to protein dissolved in 100 mM ABC. After peptide extraction in 5% formic acid (FA)/75% ACN, the liquid was evaporated under vacuum (Concentrator plus/Vacofuge plus or Concentrator 5301, Eppendorf, Nijmegen, The Netherlands) and peptides were resuspended in 20 μL of 0.1% FA.^28^

### Proteomics LC–MS Measurements

Peptides were separated and subsequently measured using an Ultimate-3000-Nano high-performance liquid chromatography system (Dionex) coupled to an Orbitrap Exploris 480 equipped with a nano-electrospray ion source (Thermo Scientific). Peptides were first chromatographically concentrated on a trap column (μPrecolumn cartridge, Acclaim PepMap100 C18, 5 μm, 100 Å, 300 μm × 5 mm, Dionex) at a flow rate of 20 μL/min and subsequently separated on a nano-LC column (Acclaim PepMap C100, C18, RSLC, 75 μm × 50 cm, 2 μm, 100 Å, nanoViper column, Dionex) at a nominal column temperature of 40 °C. A linear gradient was used for the nano-LC column at a flow rate of 0.3 μL/min from 98% of solvent A (0.1% v/v FA dissolved in Milli-Q-H_2_O) and 2% of solvent B (0.1% FA dissolved in ACN) to 45% of solvent B after 117 min. Using the μL-pickup method, 25% of each sample in the autosampler (5 °C) was injected in succession with solvent A as the transporting solvent. The mass spectrometer was set to positive ion mode and data-independent acquisition (DIA) using a precursor mass range of 300–1,200 m/z and isolation windows of 12 m/z. MS1 and MS2 resolution were set to 120,000 and 30,000, respectively.

### Data Analysis

For the analysis of ATP, LDH, and total protein content, randomized complete block design ANOVA’s 28 were conducted separately for each time point (24 h and 48 h) and treatment condition (COL (24 h), BEN (24 h), COLBEN (24 h), COL (48 h), BEN (48 h), and COLBEN (48 h)). In each analysis, the rat was the repeated/blocking unit, concentration was the fixed within-subject factor, and three technical slices were obtained per rat per concentration. A minimum of three rats were used, per analysis, with each condition repeated three times. Subsequently, pairwise post hoc tests were performed on the significant outcomes.

For proteomics data analyses, the raw LC–MS/MS data were initially processed with Spectronaut (version 17.6) (Biognosys, Schlieren, Switzerland) using default settings of the directDIA method with minor modifications. In brief, the *Rattus norvegicus* Swissprot database (http://www.uniprot.org, 8094 entries, downloaded on 02-06-2020) was used for the analysis. Only peptides with a length between 7 and 52 amino acids, with 3–6 identified MS2 fragments per peptide and no more than 2 missed cleavages, were included in the analysis. Peptide and protein group false discovery rates (FDRs) were both set to 0.01, and the maximum number of modifications per peptide was set to 5 (fixed modifications: carbamidomethyl (C), variable modifications: acetyl (protein N-term) and oxidation (methionine)). The *q*-value sparse method with the global imputing strategy was selected for quantification. Normalization across runs was subsequently performed using median scale normalization as follows:

3 $$n_{s,i}=\frac{\mathrm{dataset median}\cdot x_{s,i}}{\mathrm{sample media}n_{s}}$$

where dataset median stands for the median value of the whole dataset excluding zero values (24 or 48 h); *x_s,i_* is the value of protein *i* before normalization in sample s; *n_s,i_* is the value of protein *i* after normalization in sample *s*; and sample median*_s_* is the the median value of sample *s* excluding non-zero values.

Correction for gender effects was performed by means of linear regression, since the second rat used for the 24 h experiments was female, while the others were male rats. In the case of protein groups, the first protein of the group was seen as the representative, which protein was used in further processes representing the protein group. See Supporting Information, Data S1–S7, for an overview of all protein groups per 24 and 48 h dataset before and after normalization and gender correction. Therefore, all identified proteins, including protein groups, are named “proteins” in this paper. For comparison of differential proteins between 24 and 48 h, Venn diagrams were created with the Venny online tool.^29^ After the creation of the heatmaps and Venn diagrams, STRING analysis was performed for the 24 h data per heatmap cluster (https://string-db.org/, version 12.0).^30^ For the mapping of the data to the different functional enrichments, the background experimental dataset was set to all identified proteins in this proteomics analysis, an FDR ≤ 0.05 was used, a minimum protein count of 2 per functional enrichment was required, all active interaction sources (experiments, databases, co-expression, neighborhood, gene fusion, and co-occurrence) were included, the confidence strength was set to 0.400 (medium confidence), and disconnected nodes in the network were hidden. Most of the significant proteins could be mapped in the STRING analysis; 135 out of 136 proteins for heatmap cluster 1, 189 out of 197 proteins for heatmap cluster 2, and 93 out of 96 proteins for heatmap cluster 3. For all three heatmap clusters, the number of connections between proteins turned out to be significant compared to what would be expected from a random protein dataset (PPI enrichment *p*-values for clusters 1–3 were <1.0·10^–16^, <1.0·10^–16^, and <1.02·10^–16^, respectively). For all analyses, a *p*-value < 0.05 was considered (after correcting for multiple testing based on the FDR method) to be significant. Performing data analyses and creating figures was, except for the Venn diagrams and the String analyses, performed with R (4.2.3 (2023-03-15)). All analyses were performed on a LAPTOP-UNGDF254 with an AMD Ryzen 9 7945HX with Radeon Graphics processor running Windows 11 Home edition.

## Results and Discussion

### Experimental Design

To examine the potential effects of COL and BEN on rat PCLS, the experimental approach depicted in Figure 1 was followed. The experiments were done in a time-dependent manner (24 and 48 h), but for the 48 h experiments, COL and BEN were only added for the last 24 h to avoid causing too much damage to the PCLS. For the sake of simplicity, throughout the paper we will refer to these treatments as 24 and 48 h incubations. After incubation, viability was assessed by ATP content and LDH leakage, two endpoints known to assess the viability of rat PCLS.^25^ The ATP content reflects viability in terms of the capacity of mitochondria to generate energy from nutrients, while LDH leakage is related to the integrity of the cellular membrane. To examine tissue damage, including collagen degradation in the PCLS due to COL and BEN, histomorphology and total protein content were assessed. Specific damage of COL and BEN to all proteins was determined with proteomics.

**Figure 1:**
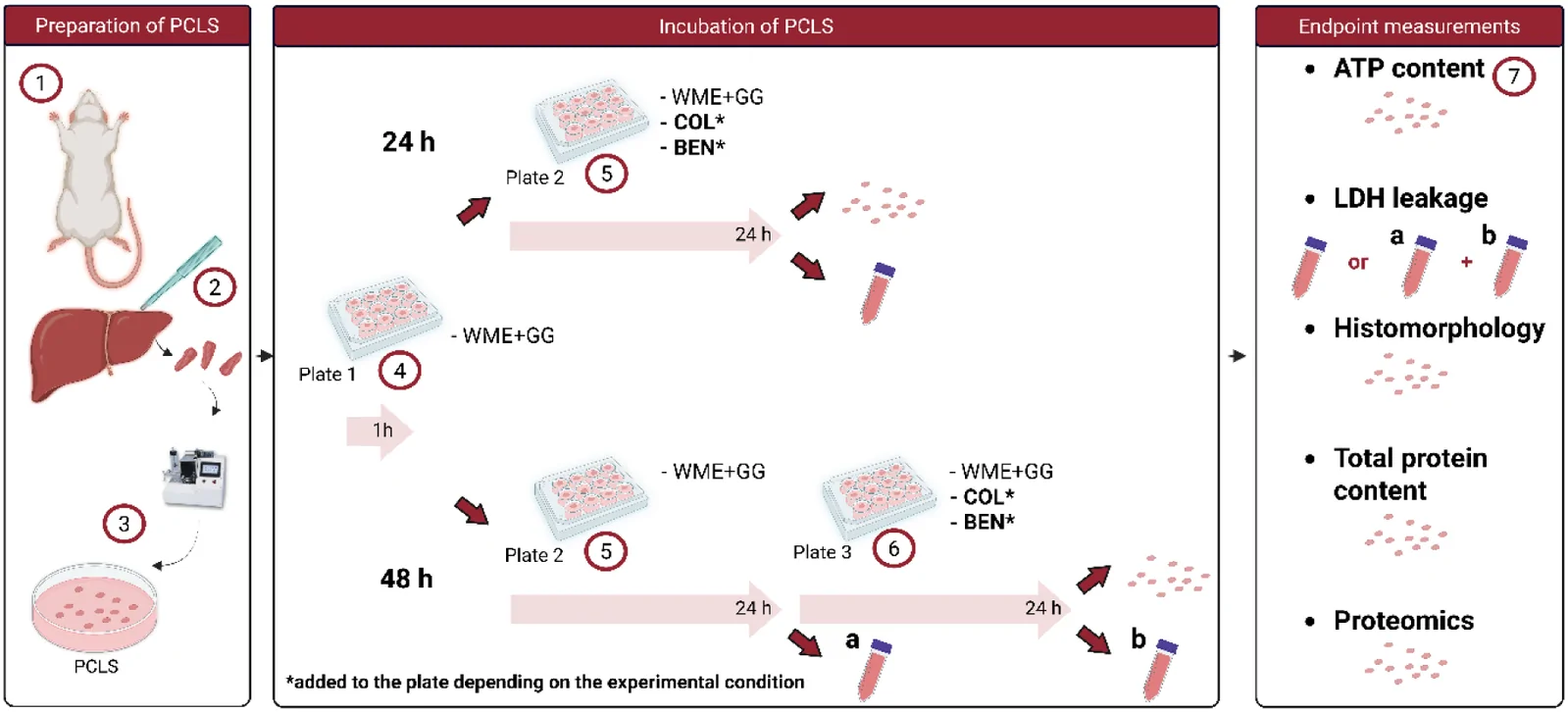
Overview of the experimental design. 1) Rats were anesthetized and the liver was removed. 2) Cylindrical cores were prepared with a biopsy punch. 3) Precision-cut liver slices (PCLS) were prepared with a Krumdieck tissue slicer. 4) PCLS were pre-incubated for 1 h in William’s Medium E supplemented with gentamicin and d-glucose (WME + GG) to allow the tissue to restore and wash away any cell debris due to the slicing. 5) PCLS were incubated for 24 h in new plates filled with WME + GG in case of the 24 h treatments and in case of the 48 h treatments also with specific enzyme(s) such as COL, BEN, or COLBEN, depending on the experimental condition. Afterward, the conditioned culture medium was stored at −80 °C. In the case of 24 h incubations, the PCLS were also harvested. 6) In the case of 48 h incubations, after the first initial 24 h incubation with WME + GG, the PCLS were again incubated for 24 h in new plates filled with WME + GG and, depending on the experimental condition, with specific enzyme(s) such as COL, BEN, or COLBEN. Afterward, the PCLS were harvested and stored separately from the conditioned culture medium. 7) PCLS were used to assess ATP content, histomorphology, and total protein content and to perform proteomic assessments. The conditioned culture medium was used to assess the LDH leakage. In the case of 48 h incubations, the conditioned culture medium, collected during 0–24 h and 24–48 h, was combined.

### Viability of Rat Precision-Cut Liver Slices after Exposure to COL and BEN

Figure 2 shows no significant effects on ATP content when rat PCLS were exposed to COL, BEN, or COLBEN. However, a decreasing trend in ATP content was seen for COL and COLBEN. This effect was most pronounced for the highest COL concentration in the co-supplement condition (COLBEN), where the average decrease in ATP content was 33.8% after 24 h and 36.8% after 48 h of incubation. It should be noted that the effect of COL treatment might also be due to the contaminations with clostripain and trypsin of the collagenase used; “COL” could therefore also be read as “COL preparation”. The higher initial severity in effect after 24 h indicates the importance of allowing the PCLS to recover from the slicing process for some time before starting the combined enzymatic treatment. For the highest COL concentration, an average decrease of 7.1% after 24 h of incubation (Figure 2A) and an average decrease of 26.3% in ATP content after 48 h of incubation (Figure 2D) were found. Taken together, this suggests that COL is the primary reason for the observed decrease in ATP content in the combined addition of COL and BEN.

**Figure 2:**
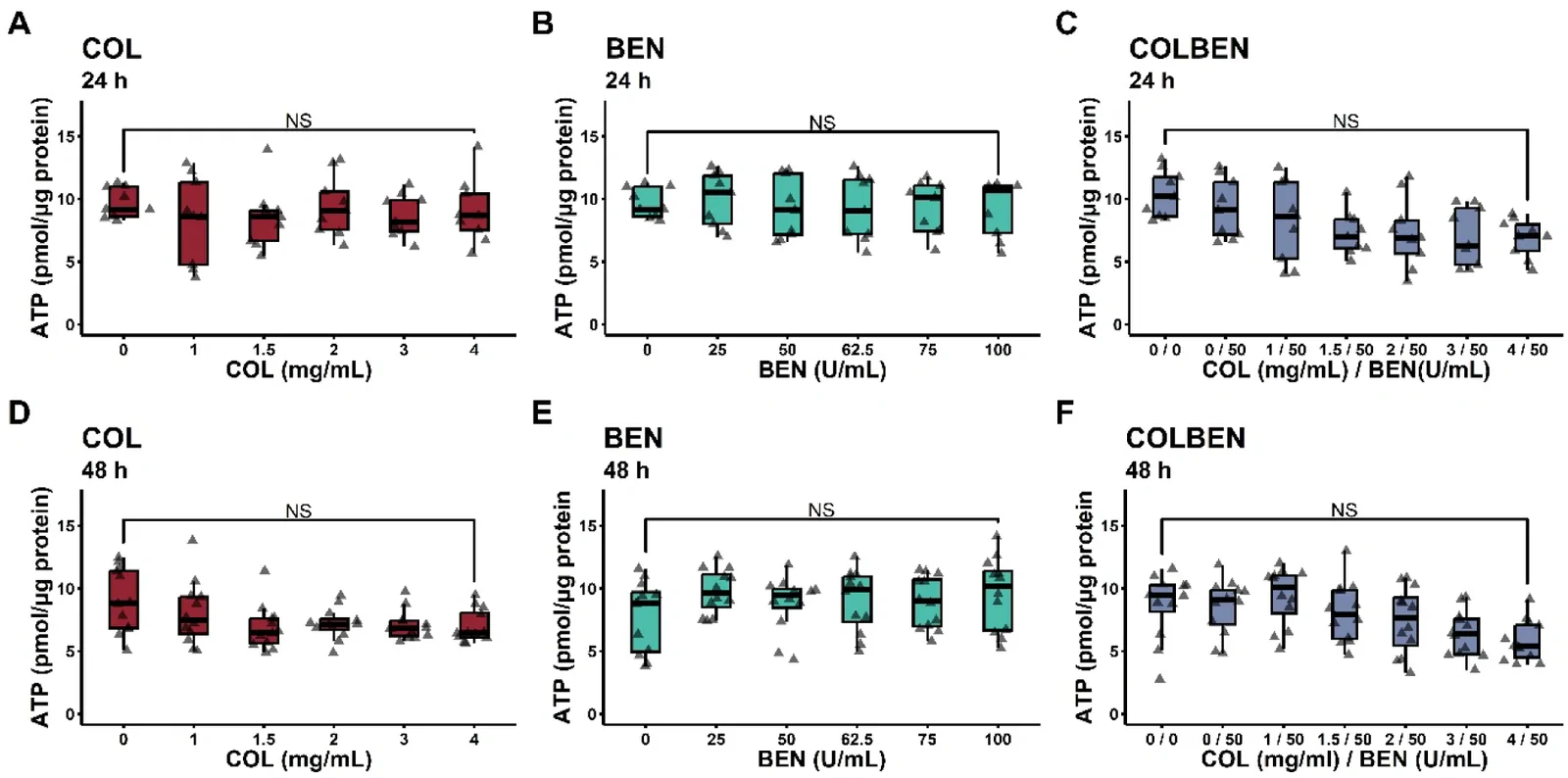
ATP content analysis of rat PCLS. ATP content is expressed in pmol/μg protein, collagenase (COL) is expressed in mg/mL, and benzonase (BEN) is expressed in U/mL. A) Different concentrations of COL after incubation of PCLS for 24 h. B) Different concentrations of BEN after incubation of PCLS for 24 h. C) Different concentrations of COL with 50 U/mL BEN (COLBEN) after incubation of PCLS for 24 h. D) Different concentrations of COL after incubation of PCLS for 48 h. E) Different concentrations of BEN after incubation of PCLS for 48 h. F) Different concentrations of COL with 50 U/mL BEN (COLBEN) after incubation of PCLS for 48 h. NS: not significant; an overall (omnibus) repeated-measures ANOVA was performed, followed by pairwise comparisons using Benjamini–Hochberg correction for multiple testing.

Figure 3 shows no significant effects for LDH leakage, although some patterns are visible, including a slight decrease in LDH leakage that was found for the highest BEN concentration (100 U/mL). This is an unexpected effect, but a possible explanation might be that BEN degrades extracellular DNA and RNA, which is detrimental for the cell membrane,^32^ but this requires further confirmation. In the combined treatment of COL and BEN, there is an increasing trend in LDH leakage between the control and increasing concentrations of COL, indicating an added effect of both treatments at high concentrations of COL (Figure 3F). Overall, it can be concluded that the LDH leakage results support the ATP results. Especially regarding the observed decreasing viability trend upon addition of COL to a fixed concentration of BEN.

**Figure 3:**
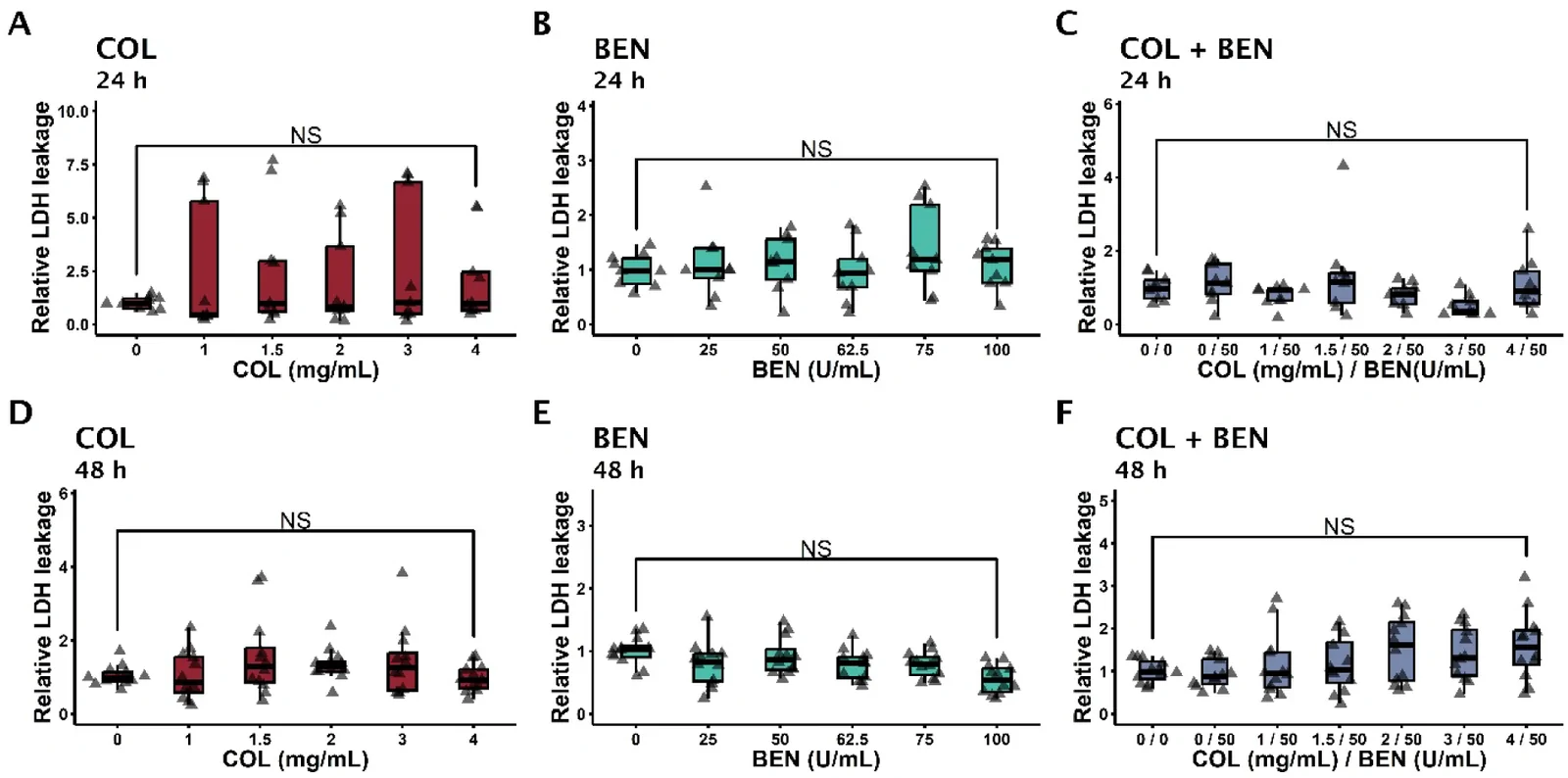
LDH leakage analysis from rat PCLS into the culture medium. Collagenase (COL) is expressed in mg/mL and benzonase (BEN) is expressed in U/mL. A) Different concentrations of COL after incubation of PCLS for 24 h. B) Different concentrations of BEN after incubation of PCLS for 24 h. C) Different concentrations of COL with 50 U/mL BEN (COLBEN) after incubation of PCLS for 24 h. D) Different concentrations of COL after incubation of PCLS for 48 h. E) Different concentrations of BEN after incubation of PCLS for 48 h. F) Different concentrations of COL with 50 U/mL BEN (COLBEN) after incubation of PCLS for 48 h. NS: not significant; an overall (omnibus) repeated-measures ANOVA was performed, followed by pairwise comparisons using Benjamini–Hochberg correction for multiple testing.

### Histomorphological Examination of Rat PCLS after Exposure to COL and BEN

To examine the morphology of rat PCLS after exposure to COL, BEN, and COLBEN, histomorphological assessments were performed after 0, 24, and 48 h of exposure (Figure 4 and Supporting File 1; Figures S1 and S2). Hematoxylin and eosin (HE) staining did not show any clear changes in overall morphology between control, COL, BEN, and COLBEN at all time points at these relatively low concentrations, indicating that no severe effects occurred due to the treatments even under conditions that affected cell viability. Also, the degree of single-cell necrosis as observed in the HE staining correlated well with the observed LDH leakage as discussed in the previous section, and no significant effects were seen between the treated conditions (Supporting Files 1; Figure S3). As expected, treatment with COL or COLBEN caused complete degradation of collagen around the central veins and portal triads as demonstrated by Sirius Red (SR) staining in Figures 4D, 4H and Supporting Files 1; Figure S2C, while treatment with BEN did not alter the collagen layer (Supporting Information 1; Figure S2D). Overall, the histomorphological analysis showed minimal morphological changes after treatment with both enzymes, consistent with the ATP and LDH results. The removal of the collagen upon COL addition (to free the EV from the ECM) was shown to be effective.

**Figure 4:**
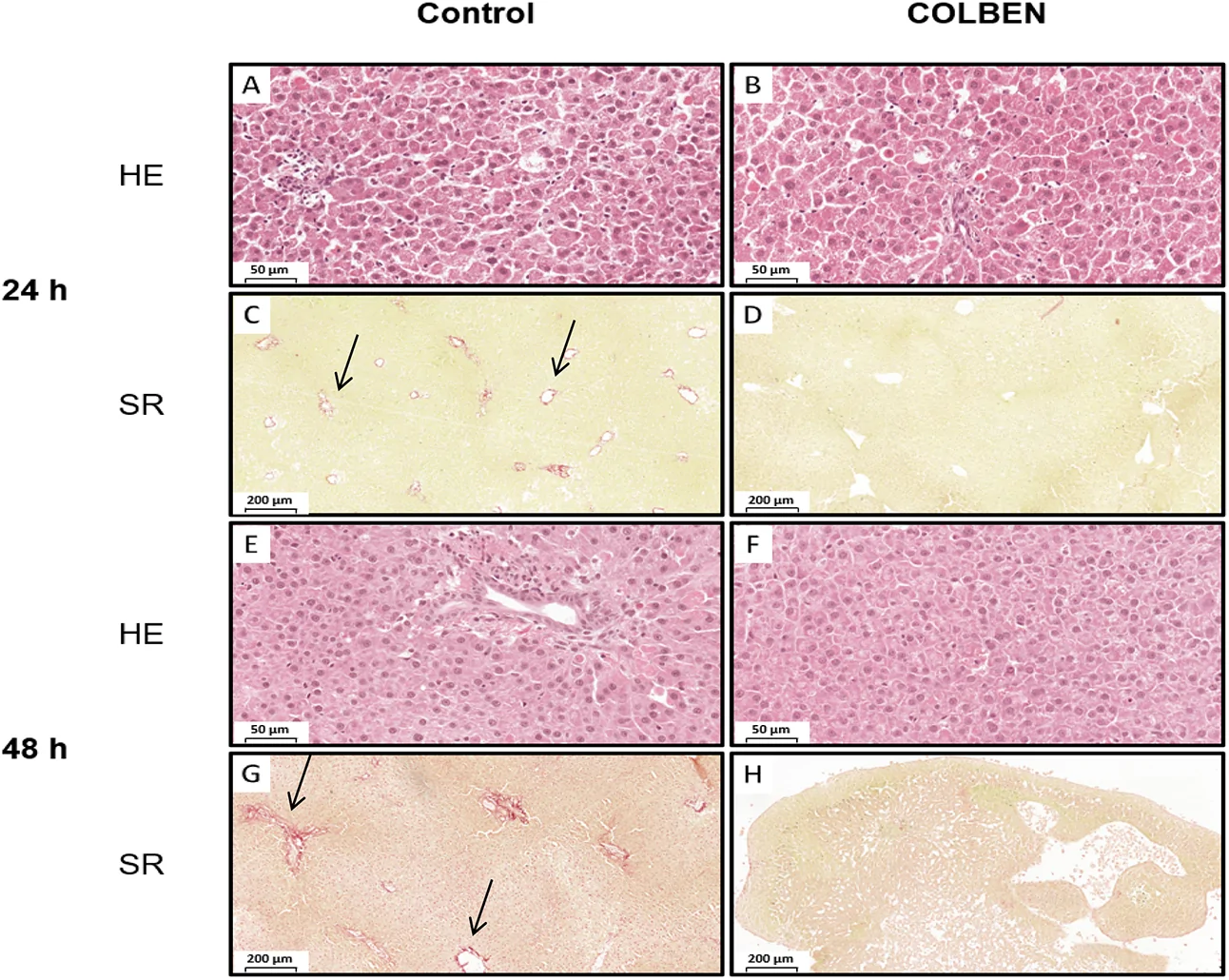
Histomorphological analysis of rat PCLS. Depicted are control rat PCLS (A, C, E, and G) and treated rat PCLS with 2 mg/mL collagenase and 50 U/mL benzonase (COLBEN) after 24 h (A and D) and after 48 h (F and H) of incubation. A, B, E, and F) Hematoxylin & eosin staining (HE). C, D, G, and H) Sirius Red staining (SR). Arrows point to examples of collagen linings since collagen is visualized in red. (See also Figures S1 and S2 in Supporting File 1 for histomorphology before incubation (0 h) and 24 h after incubation.).

### Total Protein Content Assessment of Rat PCLS after Exposure to COL and BEN

Prior to the investigation of the effects of COL and BEN on specific proteins, the effects of COL and BEN on the total protein content of rat PCLS after 24 and 48 h of incubation were assessed (Figure 5). Independent of the concentration and the incubation duration, all rat PCLS that were exposed to COL showed a minor stable decreasing trend in total protein content, although no significant effects were observed (Figure 5A and 5D). In contrast, incubation with BEN did not show this decreasing trend after 24 and 48 h of incubation (Figure 5B and 5E). When rat PCLS were exposed to different concentrations of COL as well as a fixed BEN concentration of 50 U/mL, the same trend was observed as with COL alone both after 24 and 48 h of incubation, suggesting that the presence of COL is the primary determinant for the observed minor decrease in total protein content (Figure 5C and 5F).

**Figure 5:**
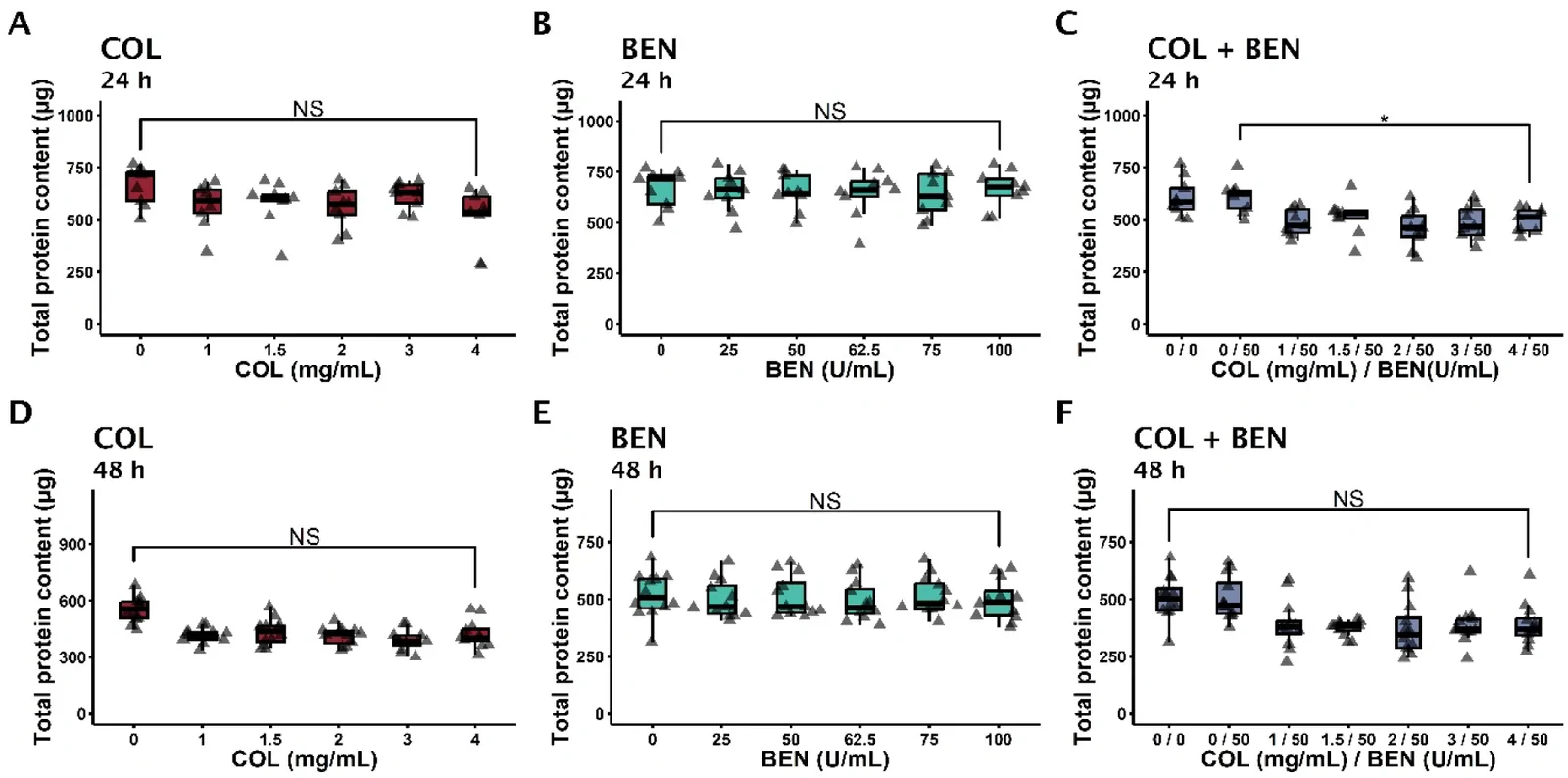
Total protein content analysis of rat PCLS. Total protein content is expressed in μg, collagenase (COL) is expressed in mg/mL, and benzonase (BEN) is expressed in U/mL. A) Different concentrations of COL after incubation of PCLS for 24 h. B) Different concentrations of BEN after incubation of PCLS for 24 h. C) Different concentrations of COL with 50 U/mL BEN (COLBEN) after incubation of PCLS for 24 h. D) Different concentrations of COL after incubation of PCLS for 48 h. E) Different concentrations of BEN after incubation of PCLS for 48 h. F) Different concentrations of COL with 50 U/mL BEN (COLBEN) after incubation of PCLS for 48 h. *P < 0.05, NS: not significant; an overall (omnibus) repeated-measures ANOVA was performed, followed by pairwise comparisons using Benjamini–Hochberg correction for multiple testing.

### Proteomics Analysis of Rat PCLS after Exposure to COL and BEN

Next, the effects of COL and BEN treatments on the rat PCLS proteome were examined. From the ATP, LDH, and protein results, a concentration of 2 mg/mL for COL and a concentration of 50 U/mL for BEN were selected. The proteomic analysis resulted in 1,706 identified proteins across the different experimental conditions, of which 429 (24 h) and 43 (48 h) were significantly different between at least two conditions based on an omnibus repeated measures ANOVA corrected for multiple testing (1% FDR) (Figure 6 and Supporting Files 1; Figure S4). Figure 6 shows that the significant differences in protein levels after 24 h can be split into three clusters: cluster 1 (136 proteins), cluster 2 (197 proteins), and cluster 3 (97 proteins) (Supporting Information, Data S8).

**Figure 6:**
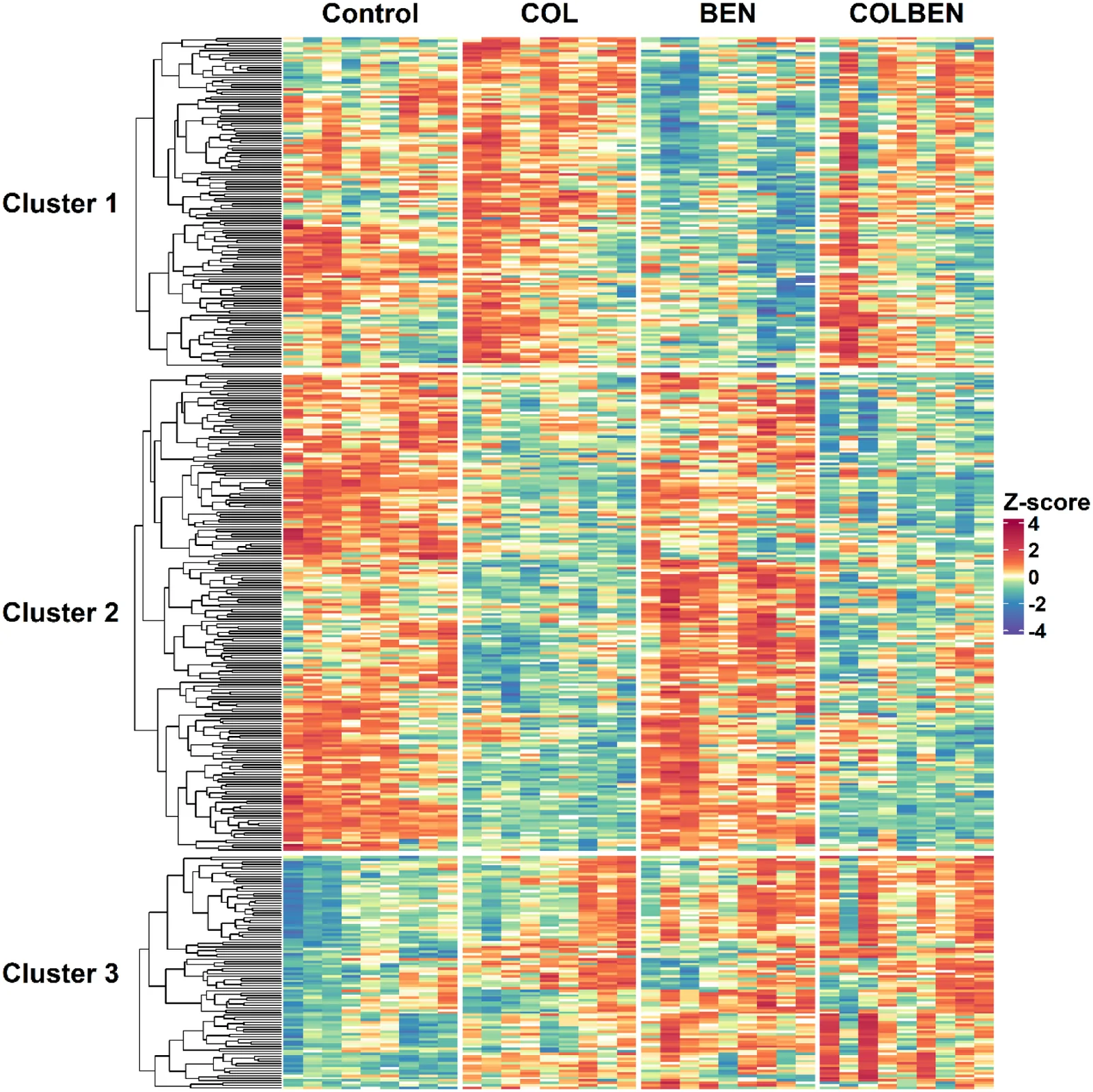
Proteomic analysis of rat PCLS incubated for 24 h. The heatmap displays z-scores of 429 significantly altered proteins after incubation of rat PCLS with or without 2 mg/mL collagenase (COL) and/or 50 U/mL benzonase (BEN). Each row represents one differential protein and each column represents one sample replicate. The more red or the more blue the color, the higher the positive or negative deviation in units of standard deviation from the mean for the particular protein. Proteins are grouped into three clusters based on a K-means approach (the full list of significant proteins in each cluster is listed in the Supporting Information, Data S8). Statistical significance was determined with ANOVA repeated measures at 1% FDR. (Abbreviations: BEN: Benzonase; COLBEN: Collagenase and Benzonase; COL: Collagenase).

Based on the heatmap, proteins belonging to cluster 1 seem to be affected by BEN treatment, proteins belonging to cluster 2 due to COL or COLBEN treatment, and proteins belonging to cluster 3 are nondiscriminant for the type of enzyme treatment. Additionally, specific pairwise comparisons (Supporting Files 1; Table S2 and Figure S4) between the different test conditions demonstrate to what extent these hypotheses can be statistically supported. Especially the cluster 2 hypothesis seems to be supported since 103 of the 197 significantly altered proteins were significant between the control and the COL condition (Supporting Information, Data S9).

Similarly, Figure S5 and S6 in Supporting Files 1 show significant proteins after 48 h of treatment of rat PCLS that can be divided into two clusters, where cluster 1 (32 proteins) seems to represent proteins significantly increased due to treatment with COL or COLBEN and cluster 2 (11 proteins) seems to represent proteins significantly decreased due to treatment with COL or COLBEN (Supporting Information, Data S10–S11).

When comparing the 24 and 48 h datasets, it is observed that almost 70% of the significantly altered proteins in the 48 h dataset (primarily cluster 1) (Supporting File 1; Figure S6) were also significantly altered in the 24 h dataset (primarily cluster 2) (Supporting File 1; Figure S6). Also, the 30 shared proteins show a similar differential up- and downregulation pattern (Supporting File 1; Figure S8). The larger response in the 24 h dataset could be due to rat PCLS being more susceptible to enzyme treatment directly after the slicing process, as they have not yet recovered from slicing. The PCLS from the 48 h datasets could recover the first 24 h from the slicing process before enzyme treatment started. As a result, more proteins from the 24 h dataset are significantly affected compared to when the enzyme treatment is started 24 h after slicing. This is also supported by the higher amount of LDH that is released into the cell culture medium during the first 24 h compared to the last 24 h (Supporting File 1; Figure S9).

### Proteomics Analysis of Rat PCLS after Exposure to COL and BEN: In-Depth Overview

Next, pathways and cellular processes represented by the affected proteins were studied in more detail, focusing primarily on the 24 h results. To do so, STRING analysis per cluster was performed to identify the interactions between proteins. Also, more insight was obtained by looking further into the relevant cellular pathways and processes of proteins that showed the clearest interaction networks. Full lists of identified cellular pathways and processes and the corresponding proteins per 24 h cluster can be found in the Supporting Information, Data S12–S15. We focused on the three identified subclusters in the 24 h dataset, since the differentially regulated proteins after 48 h mostly overlap with the proteins observed in the 24 h dataset.

Figure 7 shows that the proteins that are mainly decreased in BEN compared to COL, COLBEN, and Control (cluster 1 in Figure 6) belong to two clearly observable subclusters in the protein–protein interaction plot: protein biosynthesis and cell-cycle checkpoints. Other proteins are mainly related to the immune system or present in the cytoplasm (see Supporting Information, Data S10).

**Figure 7:**
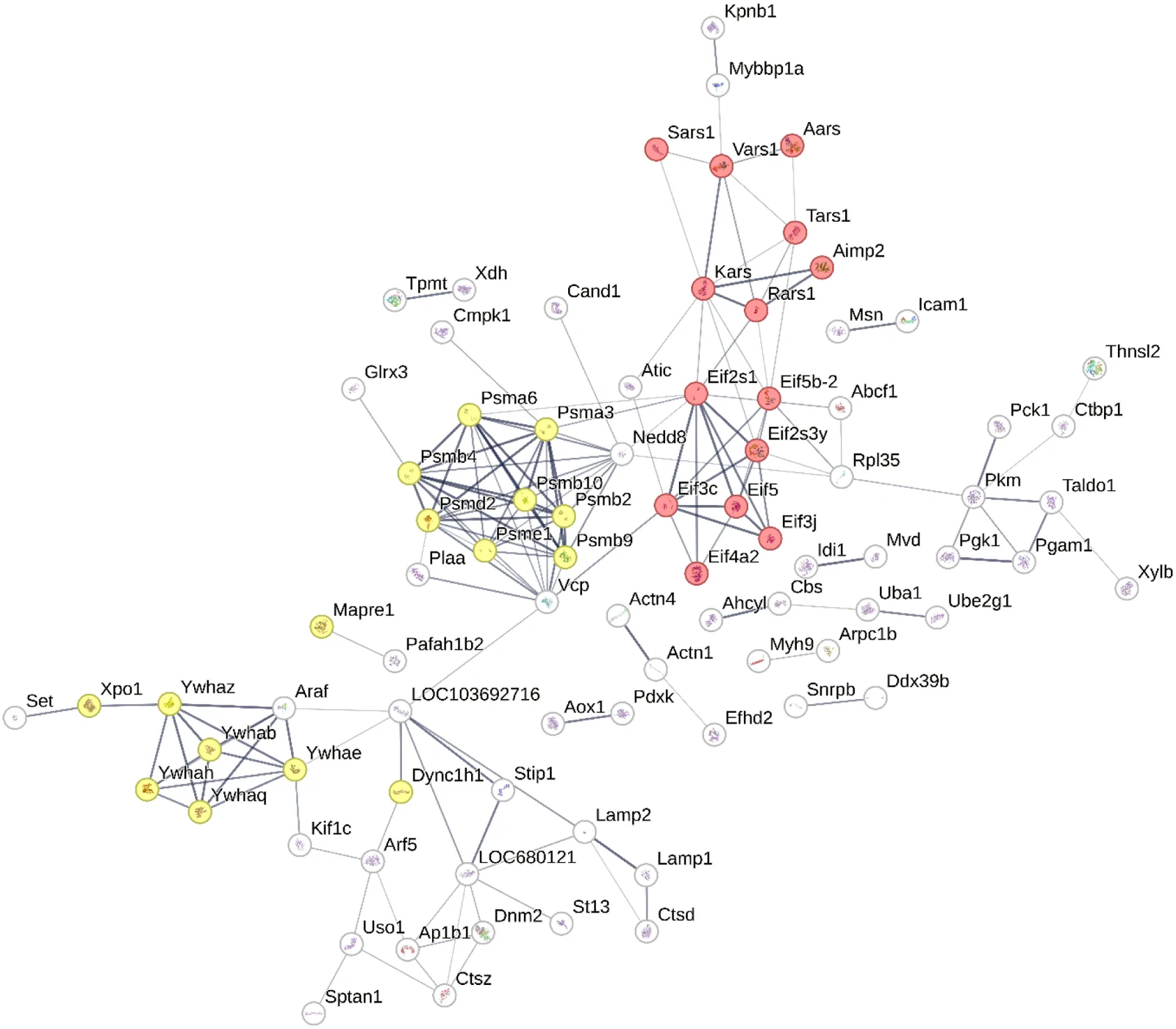
Protein-protein interaction network analysis of rat PCLS incubated for 24 h. The plot displays the STRING analysis of significantly altered proteins (cluster 1, 24 h from heatmap Figure 6). Proteins are depicted with their gene names. The thickness of the edges represents the confidence of the connection between proteins. Red subcluster: protein biosynthesis (Annotated Keywords (UniProt)). Yellow subcluster: cell cycle checkpoints (Reactome pathways). (PPI enrichment *p*-value: <1.0·10^–16^). Explanations of the gene name abbreviations are listed in Supporting Information, Data tables.

Of the 14 involved proteins in the protein biosynthesis subcluster, six proteins were aminoacyl-tRNA synthetases, which are proteins that load amino acids onto corresponding tRNAs to assist in translating mRNA to protein. This decrease is most likely a consequence of RNA degradation by the addition of benzonase. Another protein subcluster that was downregulated is based on cell-cycle checkpoints. Eight of the 16 proteins in this subcluster were proteasomal proteins, indicating that not only protein biosynthesis but also protein degradation was downregulated. This could be hypothesized to be in line with the unaffected total protein content after treatment with BEN (Figure 5), indicating that the effects of BEN on protein concentrations are only minor. The 48 h dataset does not show a cluster of proteins (Supporting File 1; Figure S6), therefore indicating that the protein alterations are only present when treating the cells directly after the slicing (24 h treatment).

Figure 8 shows that the decreased proteins from cluster 2 in Figure 6 can be divided into a few subclusters. The main subcluster contains the ribonucleoproteins. Within this subcluster, 22 ribosomal proteins were significantly decreased for COL­(BEN) compared to the control and BEN. Ribosomes can be present in the extracellular environment,^33,34^ and degradation of the extracellular matrix may have resulted in the release of these ribosomes into the culture medium.^34^ Next to ribosomes, other secreted proteins were also significantly decreased due to COL­(BEN) treatment. Examples are the collagens Col1a1 and Col1a2, which are direct substrates of COL. Other examples of secreted proteins that were downregulated were three apolipoproteins (Apoe, Apoa4, and Apoa5), which can bind to the extracellular matrix.^35^

**Figure 8:**
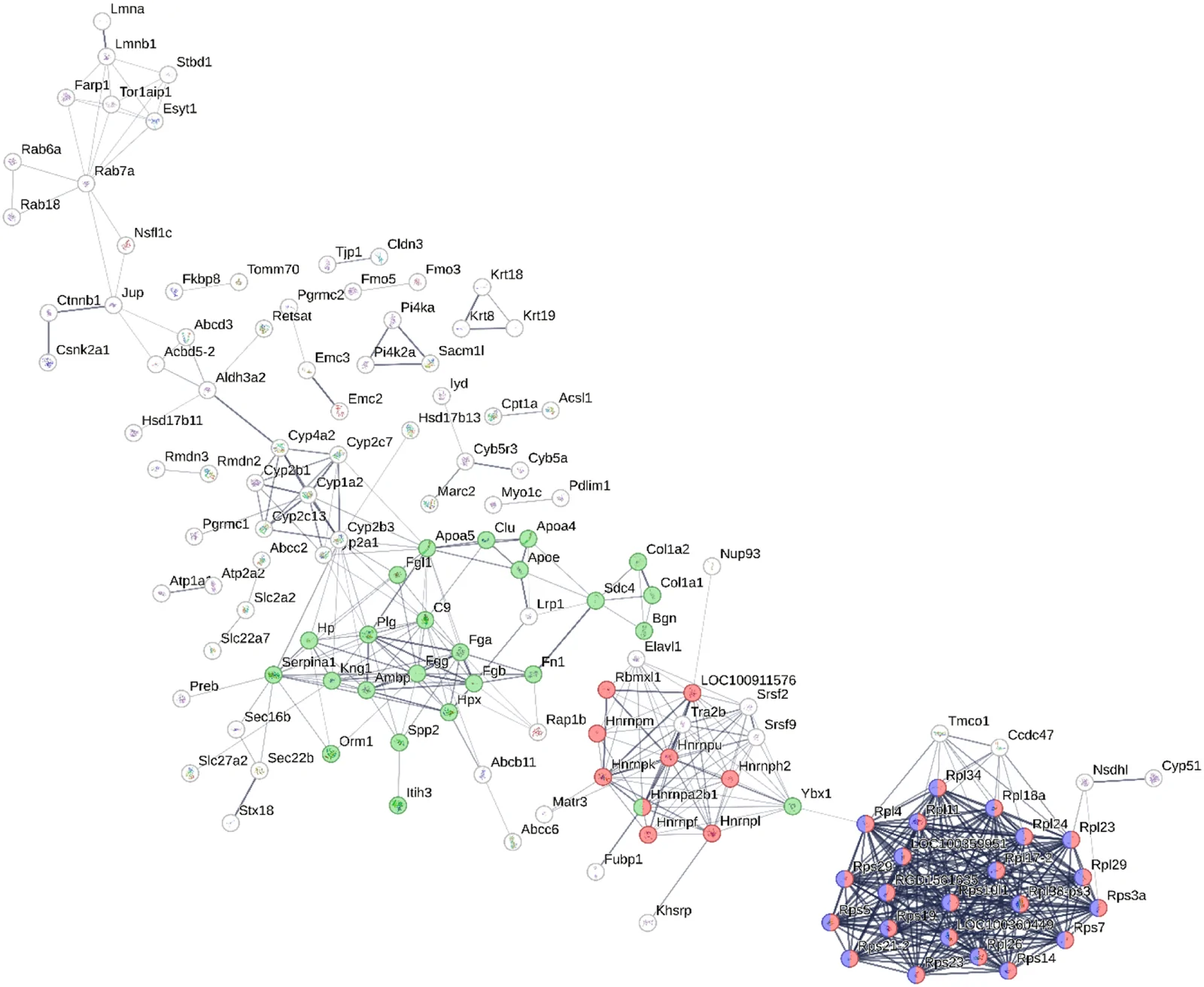
Protein-protein interaction network analysis of rat PCLS incubated for 24 h. The plot displays the STRING analysis of significantly altered proteins (cluster 2 from heatmap Figure 6). Proteins are depicted with their gene names. The thickness of the edges represents the confidence of the connection between proteins. Blue subcluster: ribosomal proteins (Annotated Keywords (UniProt)); green subcluster: secreted proteins (Annotated Keywords (UniProt)); red subcluster: ribonucleoproteins (Annotated Keywords (UniProt)). (PPI enrichment *p*-value: < 1.0·10^–16^). Explanations of the gene name abbreviations are listed in Supporting Information, Data tables.

Mapping the significantly changed proteins from the 48 h dataset to the 24 h dataset (Supporting File 1; Figure S6 and S7), several ribonucleoproteins were found, including small ribosomal subunit protein uS11 (Rps14), small ribosomal subunit protein uS7 (Rps5), heterogeneous nuclear ribonucleoprotein K (Hnrnpk), and large ribosomal subunit protein uL14 (Rpl23). Also, several secreted proteins were found, such as clusterin (Clu) and fibrinogen-like protein 1 (Fgl1). All of these were also found to be significantly altered in the 24 h dataset. In summary, these results indicate that the effects observed upon the treatment with COL­(BEN) are primarily ribosomal proteins and other secreted proteins, indicating that the ECM was (partly) broken down by COL­(BEN), causing the release of those proteins. This means that care should be taken with the biological interpretation of these proteins when studying the PCLS alongside the EV when COL­(BEN) is added for the improved release of extracellular vesicles.

Figure 9 shows the generic responses observed in the PCLS upon addition of any of the studied enzymes (cluster 3 in Figure 6). The majority of the proteins are increased due to the addition of COL, BEN, or COLBEN and belong to the main subcluster related to the mitochondrion (54 proteins), or more specifically to the mitochondrial respirasome, defined as the supercomplex of electron transport chain (ETC) complexes (12 proteins). This indicates an increased energy production,^36^ potentially to counterbalance the stress caused by COL­(BEN).^37–39^ Alternatively, it could provide energy for the activity of the upregulated proteins involved in the detoxification of COL­(BEN),^40^ including potentially the CYP P450 enzymes, of which 7 proteins belonging to the metabolism of xenobiotics by cytochrome P450 were significantly more abundant in the treated conditions versus controls.

**Figure 9:**
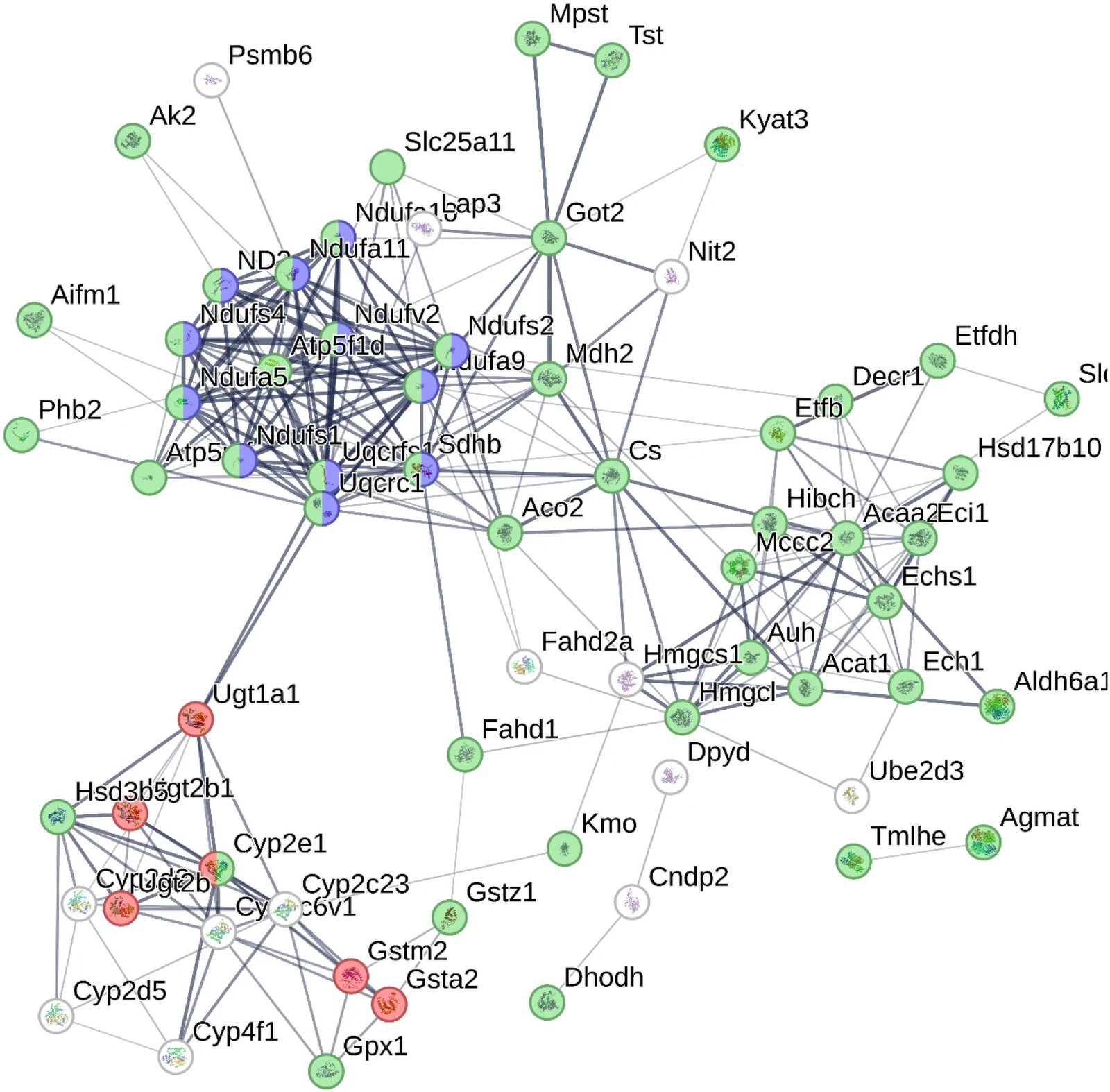
Protein-protein interaction network analysis of rat PCLS incubated for 24 h. The plot displays the STRING analysis of significantly altered proteins (cluster 3, 24 h showing proteins significantly increased due to collagenase (COL), benzonase (BEN), or collagenase and benzonase (COLBEN) treatment from heatmap Figure 6). Proteins are depicted with their gene names. The thickness of the edges represents the confidence of the connection between proteins. Blue subcluster: mitochondrial respirasome (Cellular component GO), green subcluster: mitochondrion (Cellular component GO), red subcluster: metabolism of xenobiotics by cytochrome P450 (KEGG pathways). (PPI enrichment *p*-value: < 1.0·10^–16^). Explanations of the gene name abbreviations are listed in Supporting Information , Data tables.

From the 48 h dataset, also 7 proteins belonging to the process of metabolism of xenobiotics were significantly upregulated, although not the same proteins as those in the 24 h dataset. Overall, identifying the general response of the PCLS upon the addition of COL­(BEN) results mainly in effects related to additional energy supply to counterbalance the stress caused by COL­(BEN), together with the activation of detoxification pathways.

In summary, our study identified potential cellular and tissue characteristics as well as proteome alterations of PCLS involved in the addition of collagenase and/or benzonase as a means to improve EV isolation.^11^ The addition of the enzymes had only limited effects on cell viability, although proteomics analyses do show activation of detoxification pathways and a need for increased energy production in PCLS upon addition of these enzymes. Specific effects were observed upon addition of collagenase, which breaks down collagen effectively but also seems to affect other proteins linked to the ECM, which are likely secondary effects of the collagen breakdown. Benzonase may trigger a rebalancing of protein synthesis and degradation as a consequence of its RNase activity. A limitation of the study is that the conditioned culture medium was not characterized. As a result, direct effects of COL and BEN cannot be fully distinguished from secondary effects arising from enzyme-induced changes in the extracellular microenvironment. In addition, the causality between observed effects and COL and BEN addition was not examined in detail. We cannot be fully certain that the observed effects are specific to collagenase or benzonase since both enzymes were not added as pure enzymes. The observed effects could theoretically be derived from the small percentage of coenriched other enzymes. However, the current choices reflect the relevant practical use similar to conditions chosen in experiments with tissue-derived starting materials.^22^

In conclusion, our measurements show that the addition of the enzymes has distinct effects on several pathways, which could affect the biological interpretation when liver diseases are studied. Our study shows that control experiments are essential in future studies, and we provide the leads for the affected pathways. In future experiments, we want to use the optimized concentrations of both enzymes (highest concentrations with least side effects) to see how they are affecting the EVs, which was not feasible for the full range of conditions since several slice experiments need to be pooled for each EV isolation.

## Supplementary Material





## Data Availability

The mass spectrometry proteomics data have been deposited to the ProteomeXchange Consortium via the PRIDE^31^ partner repository with the dataset identifier PXD070296 and can be accessed via https://www.ebi.ac.uk/pride/archive/projects/PXD070296.

## Notes

Histomorphological analysis of the rat PCLS at 0 h­(Figure S1); histomorphological analysis of the rat PCLS at 24 h­(Figure S2); linear relation between LDH leakage and single cell necrosis at 24 h­(Figure S3); individual volcano plot at 24 h­(Figure S4); individual volcano plots at 48 h­(Figure S5); heatmap at 48 h­(Figure S6); Venn diagrams differential regulation at 24 and 48 h­(Figure S7); heatmap Top30 differentially regulated proteins at 24 and 48 h­(Figure S8); LDH leakage at 0–24 h and 24–48 h­(Figure S9); detailed information on collagenase D batches­(Table S1); overlap between the heatmap clusters and pairwise analyses at 24 h (Table S2) (PDF)
All full tables with the identified proteins and post-analyses steps (Data S1–S15) (XLSX)
