# Supplementary material for: Toxicological Exploration of Collagenase and Benzonase on Rat Precision-Cut Liver Slices
Source: J Proteome Res. 2026 Sep 17;25(10):5049–61. doi: 10.1021/acs.jproteome.5c01232 (PMC13640514; doi:10.1021/acs.jproteome.5c01232)
Supplement: Supplementary file 1 [file pr5c01232_si_001.pdf]

Toxicological exploration of collagenase and benzonase on rat precision-cut liver slices

Janine Stam,[†] Peter Olinga,[‡] Aswin Menke,[§] Pieter Annaert,[ǁ,⊥] Klaas Nico Faber,[#] Peter L. Horvatovich,[†] Rainer Bischoff,[†] Justina C. Wolters[*,∇]

[†] Department of Analytical Biochemistry, Groningen Research Institute of Pharmacy, University of Groningen, Groningen 9700 AD, The Netherlands

[‡] Department of Pharmaceutical Technology and Biopharmacy, Groningen Research Institute of Pharmacy, University of Groningen, Groningen 9713 AV, The Netherlands

[§] Department of Metabolic Health Research, The Netherlands Organization for Applied Scientific Research (TNO), Leiden 2333 BE, The Netherlands

[ǁ] Drug Delivery and Disposition, Department of Pharmaceutical and Pharmacological Sciences, KU Leuven, Leuven B-3000, Belgium

[⊥] BioNotus CommV, Niel 2845, Belgium

[#] Department of Gastroenterology and Hepatology, University of Groningen, University Medical Center Groningen, Groningen 9713 GZ, The Netherlands

[∇] Department of Pediatrics, University of Groningen, University Medical Center Groningen, Groningen 9713 AV, The Netherlands

* Email: j.c.wolters@umcg.nl

Table of contents

Figure S1. Histomorphological analysis of the rat PCLS at 0 h
Figure S2. Histomorphological analysis of the rat PCLS at 24 h
Figure S3. Linear relation between LDH leakage and single cell necrosis at 24 h
Figure S4. Individual volcano plots at 24 h
Figure S5. Individual volcano plots at 48 h
Figure S6. Heatmap at 48 h
Figure S7. Venn diagrams differential regulation at 24 h and 48 h
Figure S8. Heatmap Top30 differentially regulated proteins at 24 h and 48 h
Figure S9. LDH leakage at 0-24 h and 24-48 h
Table S1. Detailed information collagenase D batches
Table S2. Overlap between the heatmap clusters and pairwise analyses 24 h
Data S1-S15. All full tables with the identified proteins and post-analyses steps

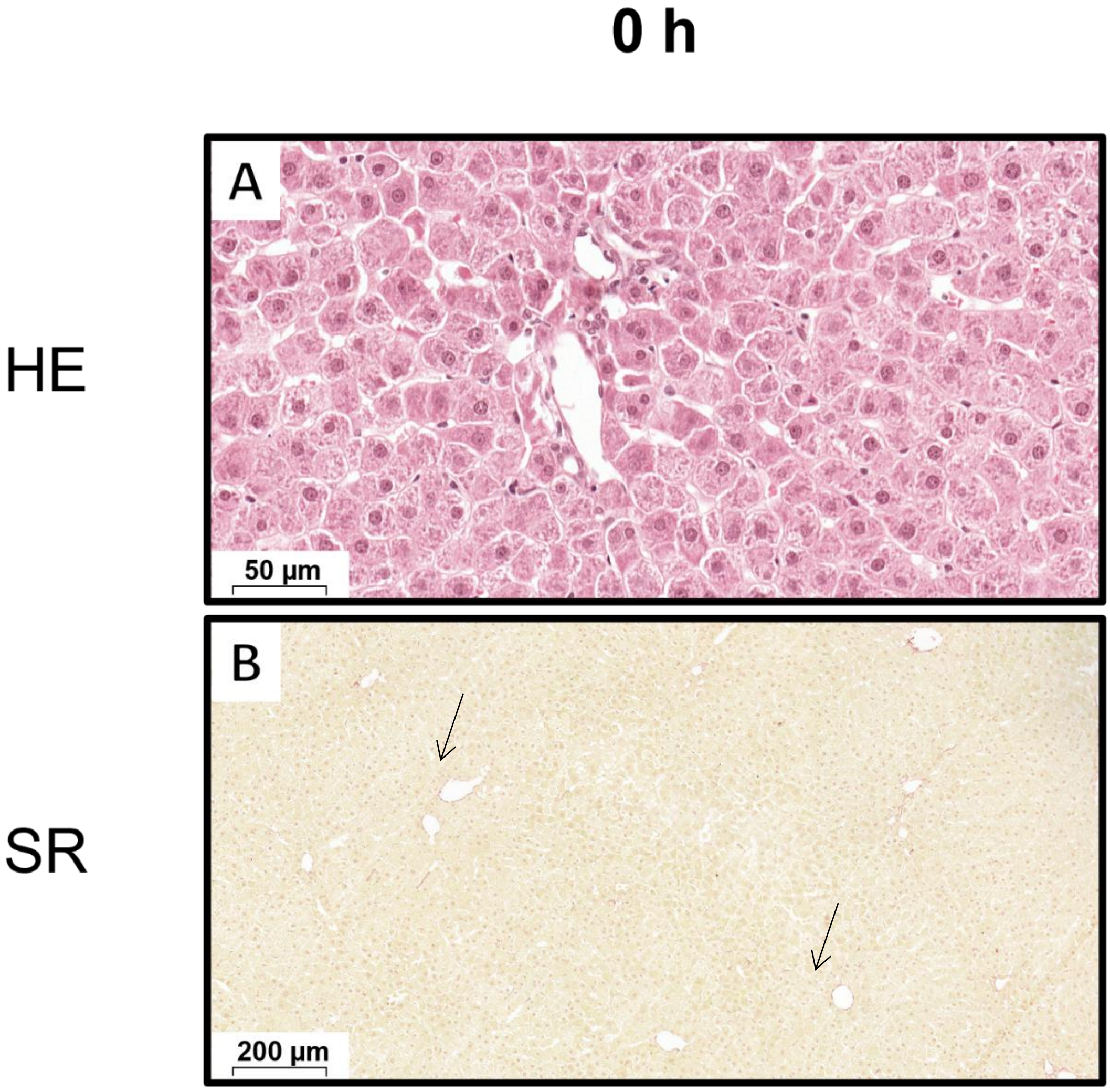

Figure S1: Histomorphological analysis of rat PCLS before incubation (0 h). A) Hematoxylin & eosin staining (HE). B) Sirius Red staining (SR). *Arrows point to examples of collagen linings since collagen is visualized in red.*

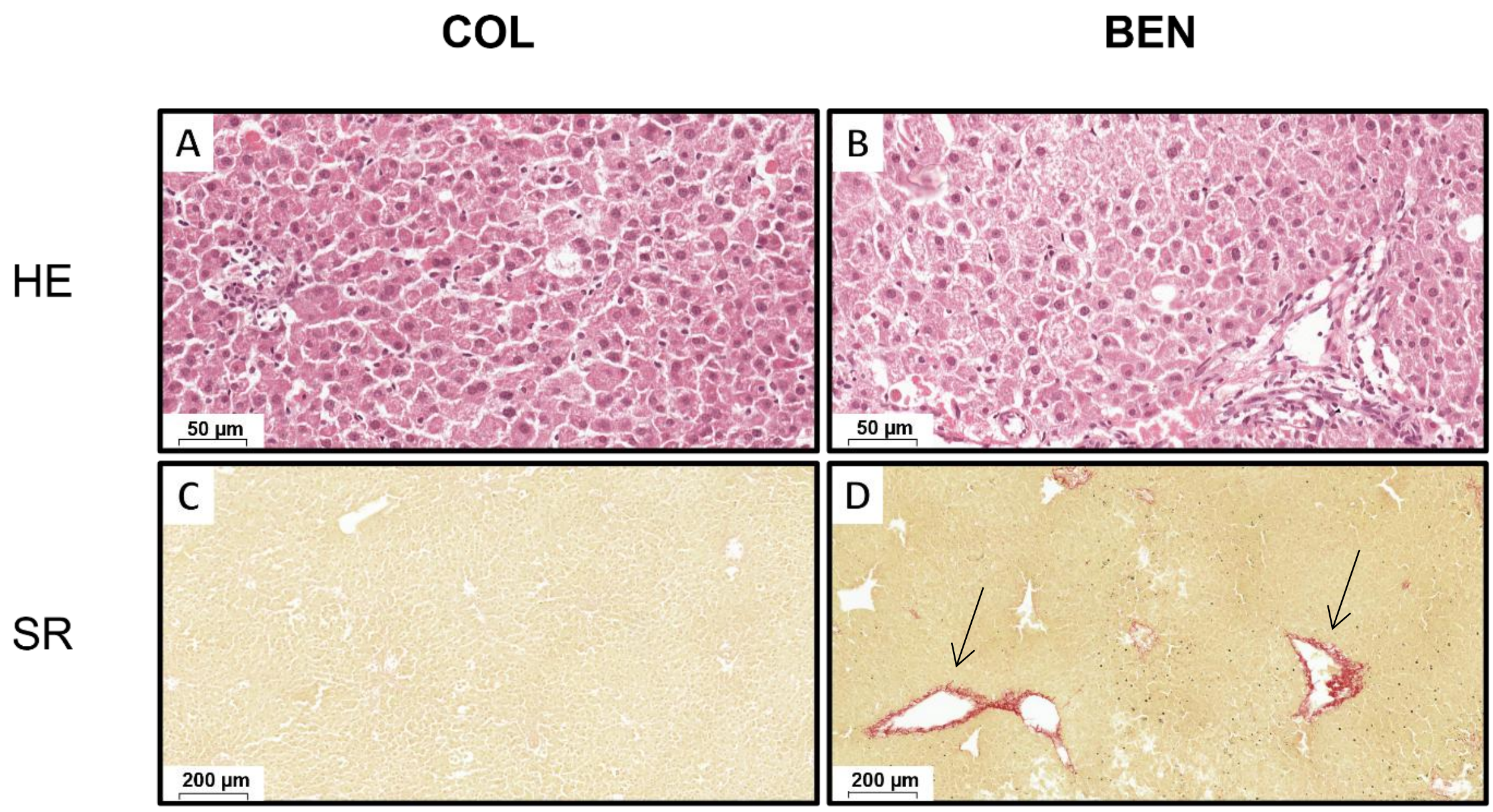

Figure S2: Histomorphological analysis of rat PCLS after 24 h of incubation. A & B) Hematoxylin & eosin staining (HE) after incubation with 2 mg/ml collagenase (COL) or 50 U/ml benzonase (BEN), respectively. C & D) Sirius red staining (SR) after incubation with COL or BEN, respectively. *Arrows point to examples of collagen linings since collagen is visualized in red.*

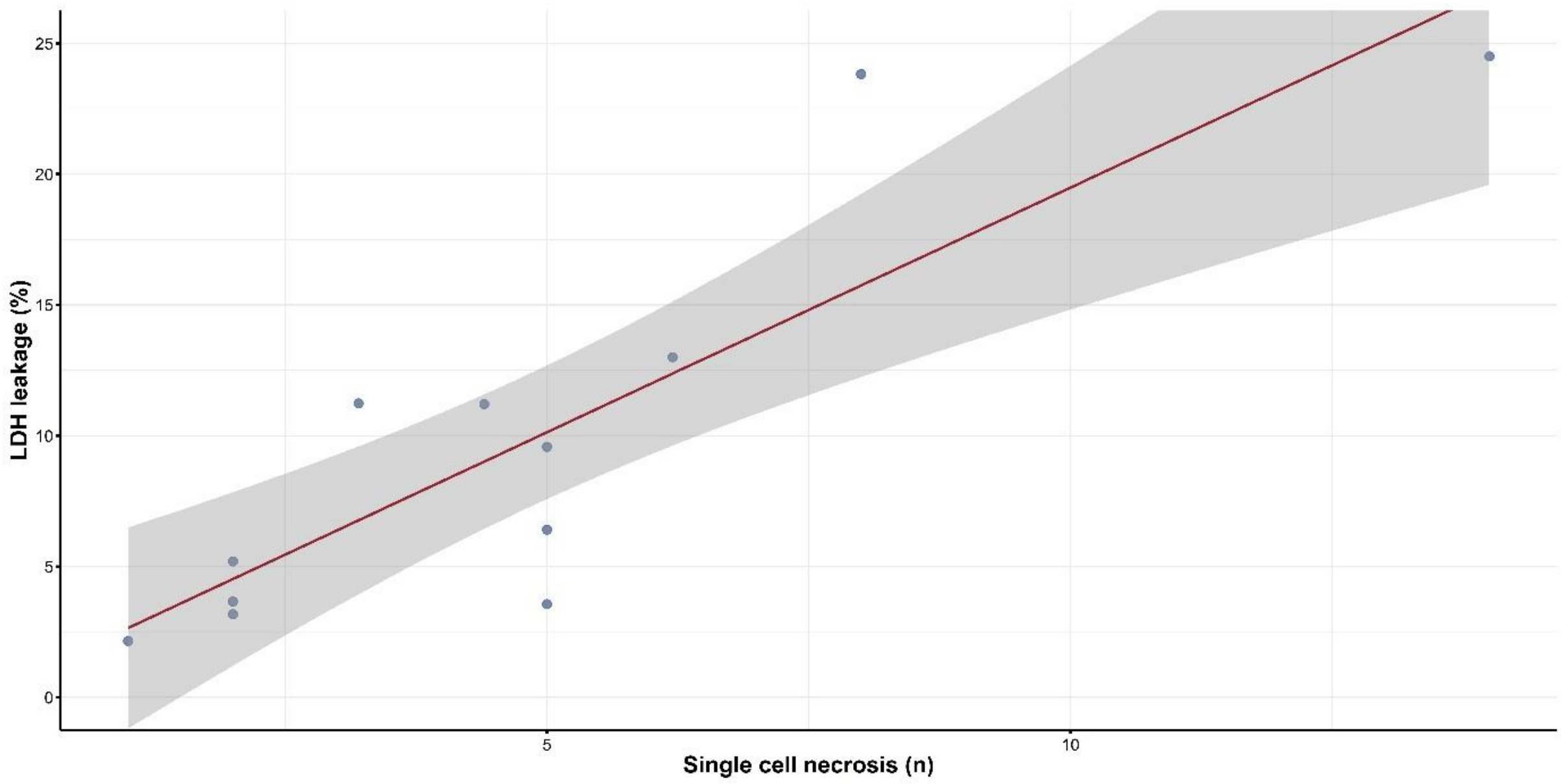

Figure S3: Linear relationship between LDH leakage and single cell necrosis in rat PCLS after 24 h of incubation. The solid red line shows the fitted linear regression (±95% confidence interval). A significant positive correlation was observed (y = 0.87 + 0.40x; R^2 = 0.87, 95% CI [0.59, 0.96]; p = 0.0002494; n = 12).

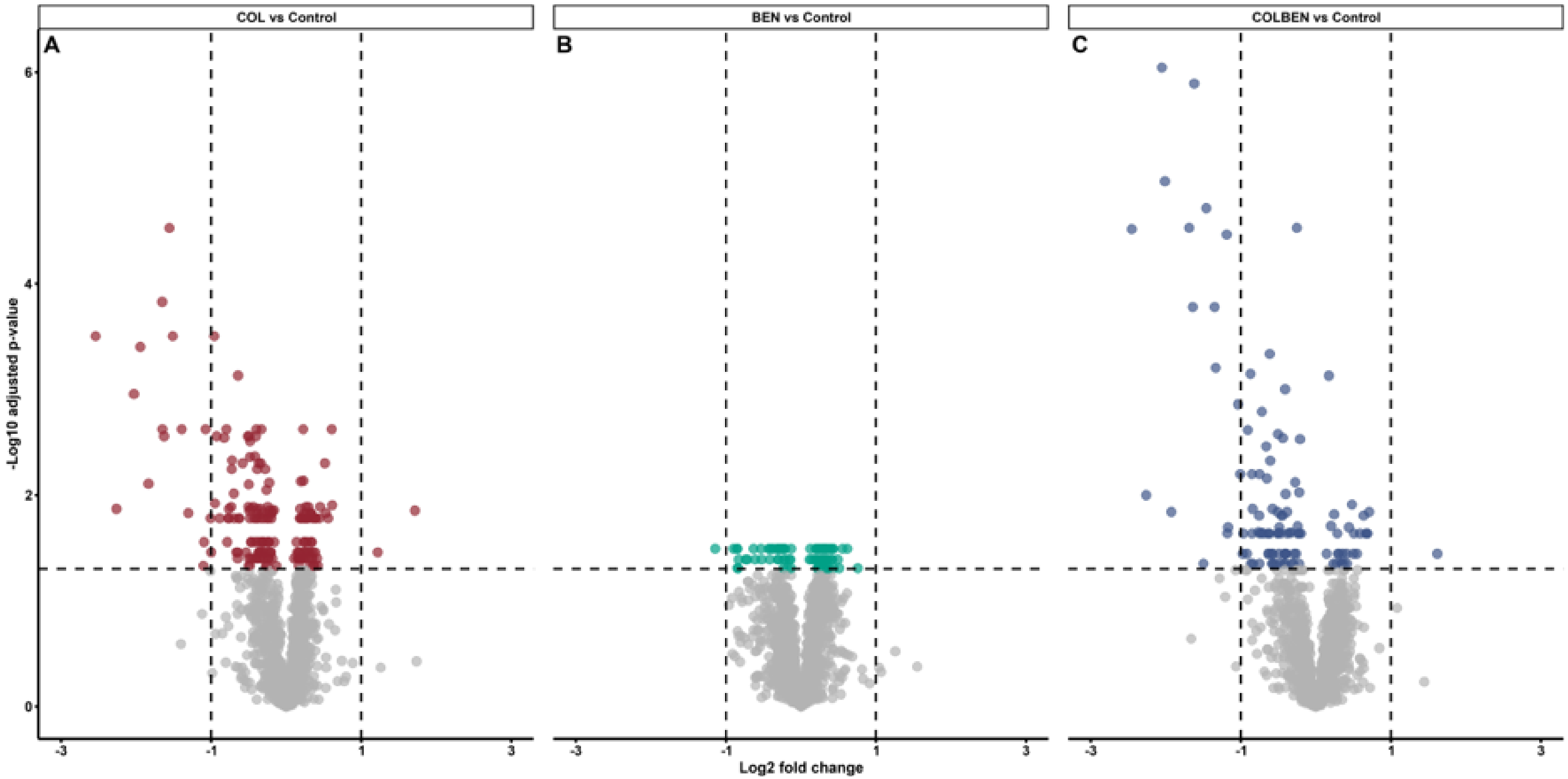

Figure S4: Volcano plots showing differential protein abundance following 24 h enzyme treatment. Volcano plots display the log2 fold change (x-axis) versus the −log10 adjusted p-value (y-axis) for proteins comparing (A) COL versus Control, (B) BEN versus Control, and (C) COLBEN versus Control. Log2 fold changes were calculated from paired differences between treatment and control conditions for each rat. Adjusted p-values were obtained from the corresponding pairwise comparisons and corrected for multiple testing. The dashed horizontal line indicates an adjusted p-value of 0.05, while the vertical dashed lines indicate log2 fold changes of −1 and +1. Significant proteins (adjusted p-value < 0.05) are highlighted in the respective treatment color; non-significant proteins are shown in grey. *(Abbreviations: BEN: Benzonase; COLBEN: Collagenase & Benzonase; COL: Collagenase).*

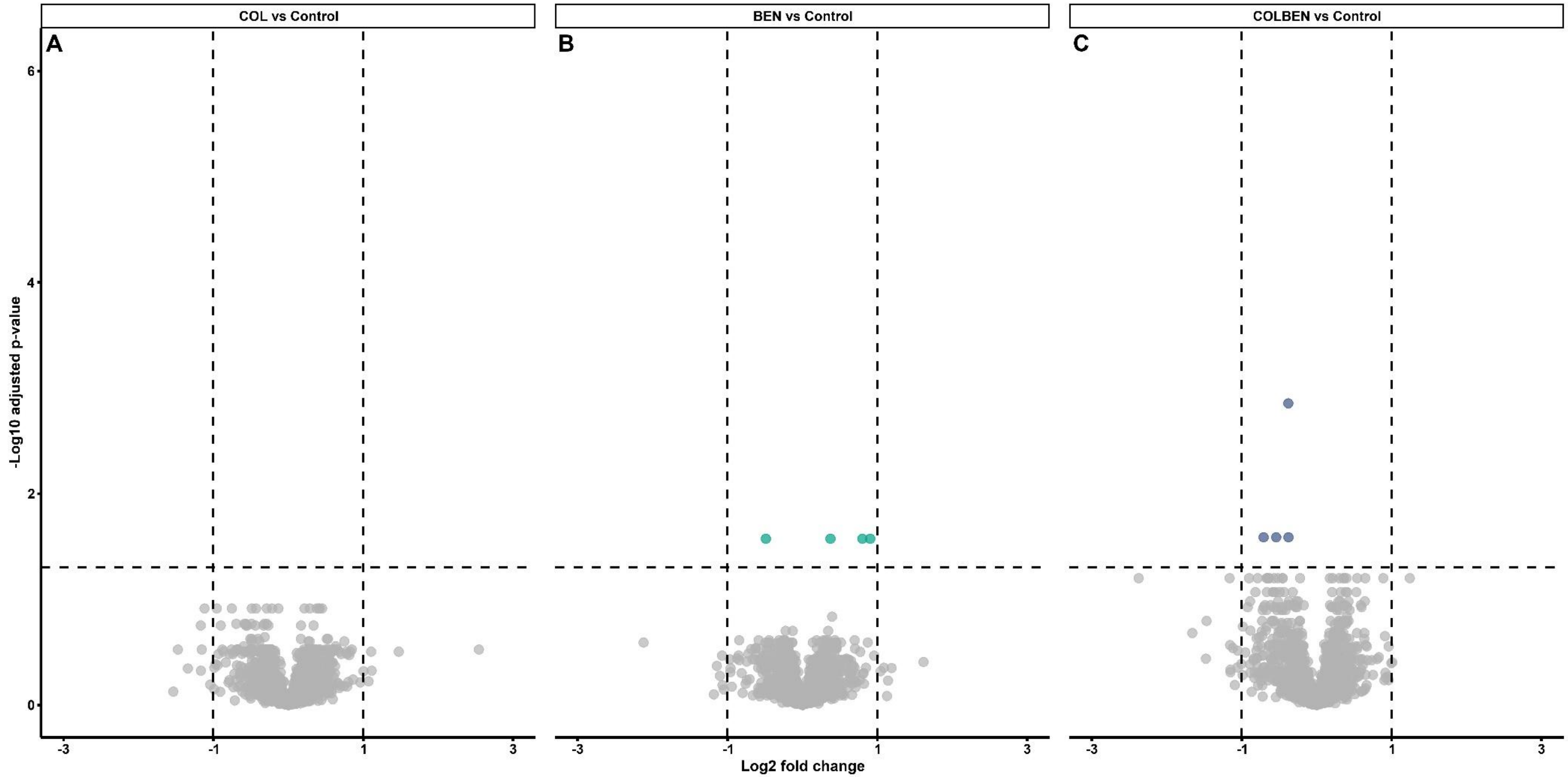

Figure S5: Volcano plots showing differential protein abundance following 48 h enzyme treatment. Volcano plots display the log2 fold change (x-axis) versus the −log10 adjusted p-value (y-axis) for proteins comparing (A) COL versus Control, (B) BEN versus Control, and (C) COLBEN versus Control. Log2 fold changes were calculated from paired differences between treatment and control conditions for each rat. Adjusted p-values were obtained from the corresponding pairwise comparisons and corrected for multiple testing. The dashed horizontal line indicates an adjusted p-value of 0.05, while the vertical dashed lines indicate log2 fold changes of −1 and +1. Significant proteins (adjusted p-value < 0.05) are highlighted in the respective treatment color; non-significant proteins are shown in grey. *(Abbreviations: BEN: Benzonase; COLBEN: Collagenase & Benzonase; COL: Collagenase).*

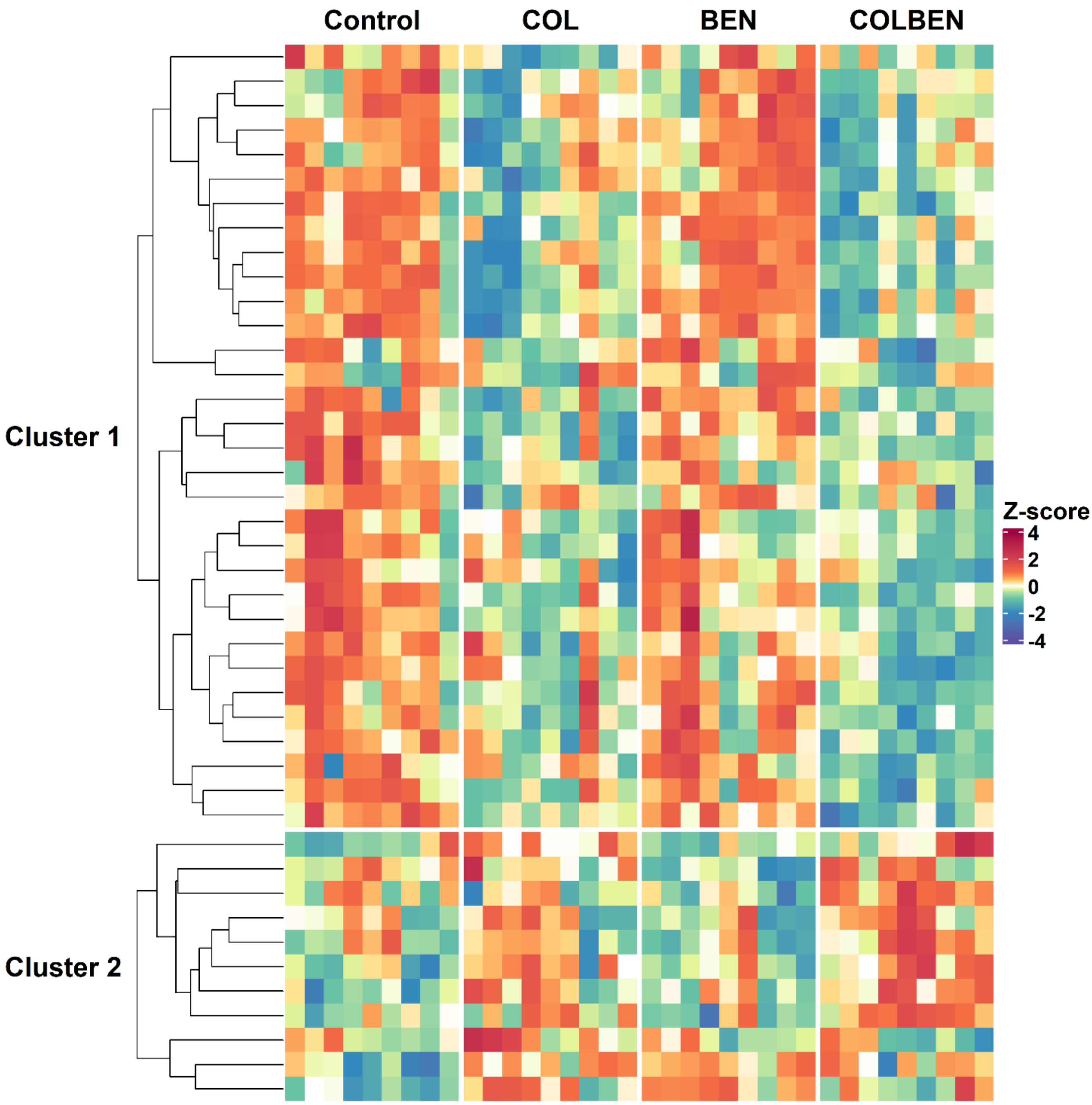

Figure S6: Proteomic analysis of rat PCLS incubated for 48 h. The heatmap displays z-scores of 43 significantly altered proteins after incubation of rat PCLS with or without 2 mg/ml COL and/or 50 U/ml BEN. (See Materials & Methods section for more details). Each row represents a differential protein, and each column represents a sample replicate. The more red or the more blue the color, the higher the positive or negative deviation from the mean in units of standard deviation for a particular protein. Proteins are grouped in two clusters based on a K-means approach. Statistical significance was determined with ANOVA repeated measures at 1% FDR. *(Abbreviations: BEN: Benzonase; COLBEN: Collagenase & Benzonase; COL: Collagenase).*

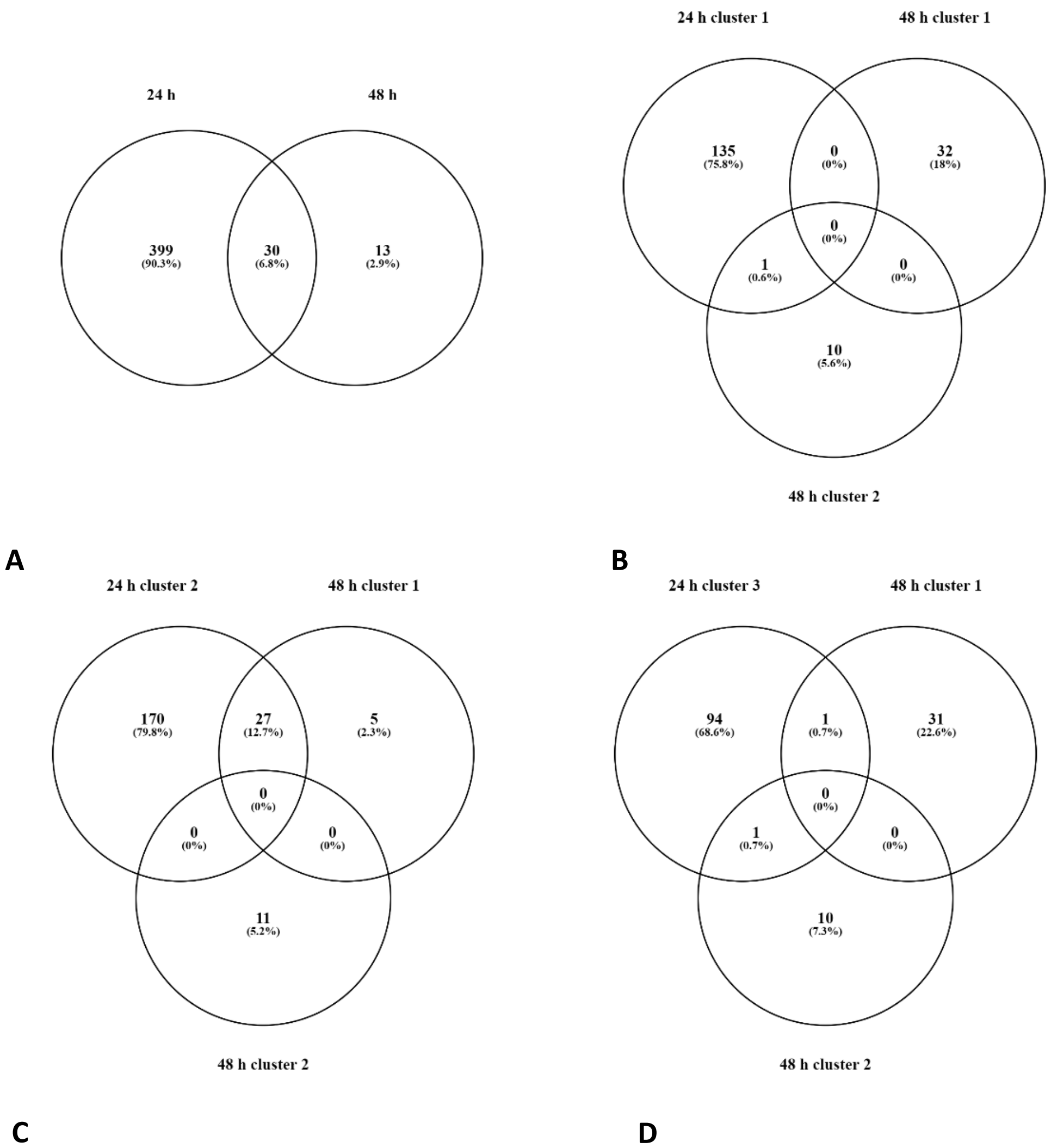

Figure S7: Overlap of differential proteins between 24 h and 48 h shown in Venn diagrams. A) Overlap between full 24 h data set and full 48 h data set. B) Overlap between the first cluster of the 24 h data set and the first and second clusters of the 48 h data set. C) Overlap between the second cluster of the 24 h dataset and the first and second clusters of the 48 h dataset. D) Overlap between the third cluster of the 24 h dataset and the first and second clusters of the 48 h dataset.

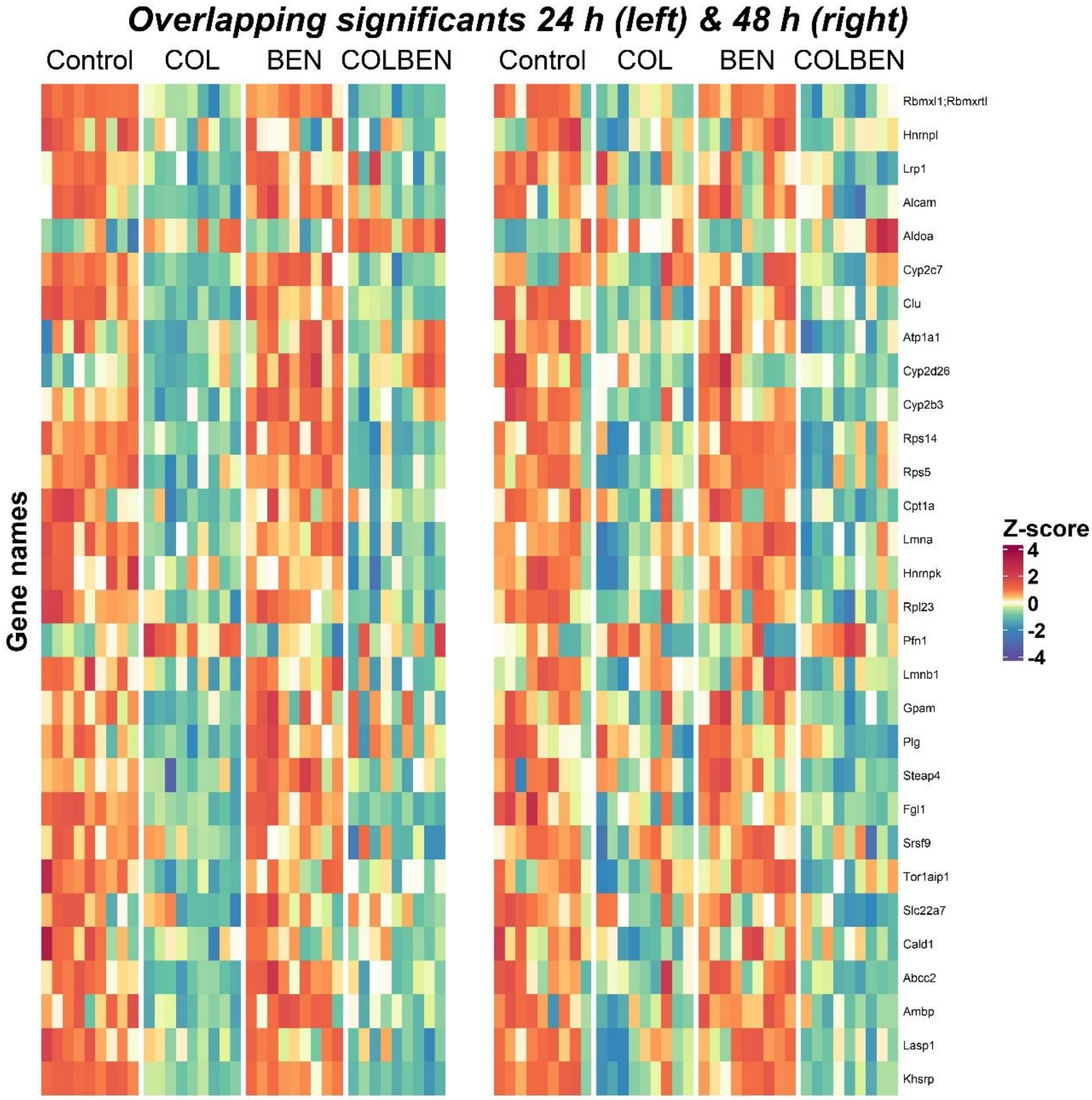

Figure S8: Proteomics analysis of rat PCLS incubated for 24 h and 48 h. The heatmaps display z-scores of 30 significantly altered proteins (corresponding gene names are shown) after incubation of rat PCLS with or without 2 mg/ml COL and/or 50 U/ml BEN for 24 h and 48 h. (See Materials & Methods section for more details). Left heatmap: z-scores for 24 h data. Right heatmap: z-scores for 48 h data. Each row represents a differential protein, and each column represents a sample replicate. The more red or the more blue the color, the higher the positive or negative deviation from the mean in unite of standard deviation for a particular protein. Statistical significance was determined with ANOVA repeated measures at 1% FDR. *(Abbreviations: BEN: Benzonase; COLBEN: Collagenase & Benzonase; COL: Collagenase). Explanations of the gene name abbreviations are listed in Supplemental files 2.*

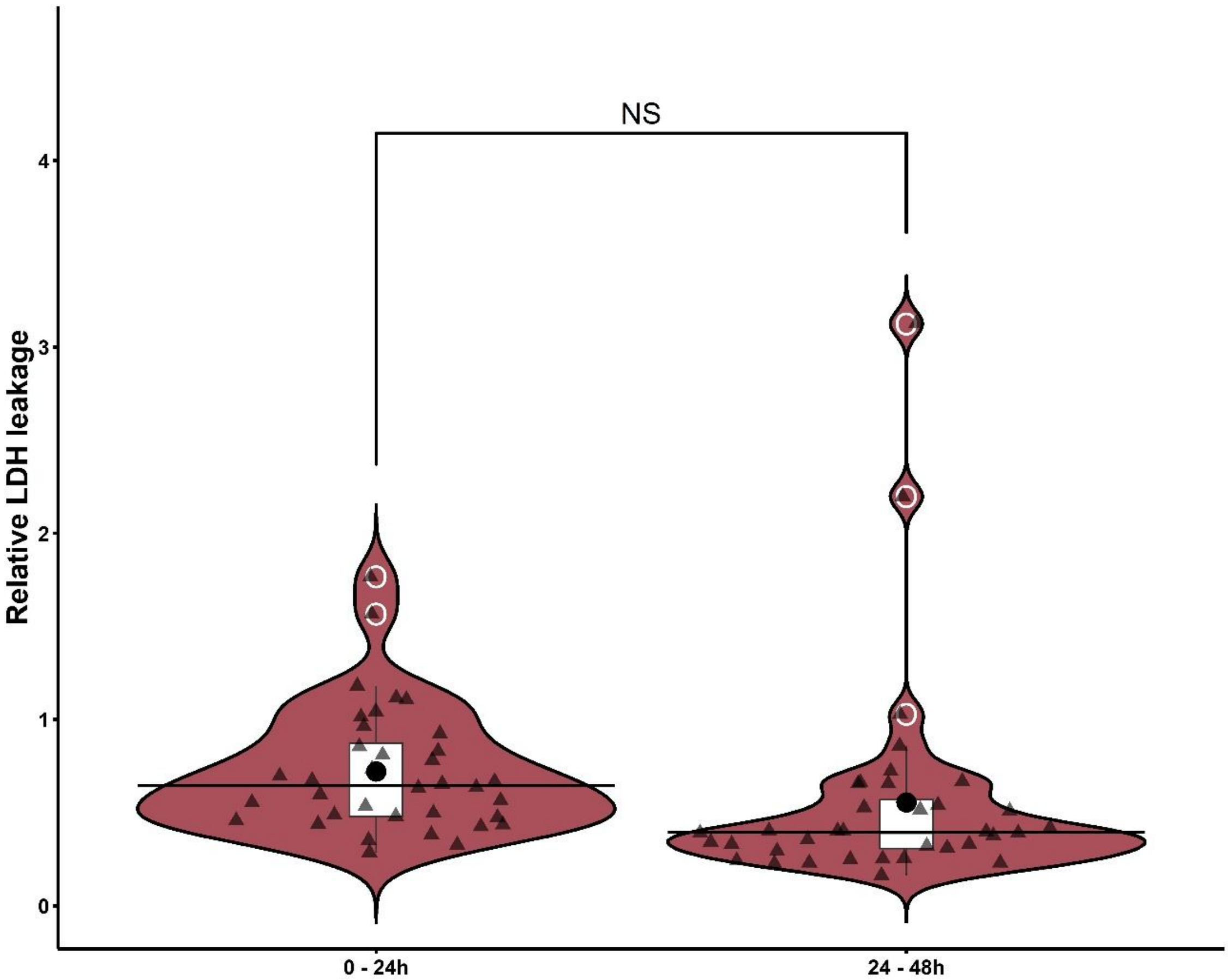

Figure S9: Relative LDH leakage from naive rat PCLS after incubation for 0 - 24 h and 24 - 48 h into the culture medium. Small black triangles (▲) depict individual values. The mean value is represented by a black dot (●) and the median value by the crossbar (▬▬). The white open circles represent outliers (◙). NS: not significant. The independent T-test resulted in a P-value of 0.1352.

Table S1: Overview of the used collagenase D batch numbers per experiment and their contaminations with clostripain, trypsin and other unspecified proteases.

Batch number	Collagenase D* (u/mgL)	Clostripain (u/mgL)	Trypsin (u/mgL)	Other unspecified proteases (u/mgL)	Used in number of individual experiments:
47895021	0.26	0.746	0.1	<1	ATP 24 h (3)<br>LDH 24 h (3)<br>Protein 24 h (3)<br>Histomorphology 24 h (3)<br>Proteomics 24 h (3)<br><br>ATP 48 h (2-3)<br>LDH 48 h (2-3)<br>Protein 48 h (2-3)<br>Histomorphology 48 h (1)<br>Proteomics (1)
46469225	0.27	1.100	<0.1	4	ATP 48 h (1)<br>LDH 48 h (1)<br>Protein 48 h (1)<br>Histomorphology 48 h (2)<br>Proteomics 48 h (2)

***Collagenase D is prepared from Clostridium Histolyticum by filtration and ammonium precipitation

Table S2: Overlap between heatmap clusters (Figure 6) and pairwise comparisons analyses from the 24 h data.

	Total number/cluster	Control – COL	Control – BEN	Control – COLBEN	COL - BEN	COL - COLBEN	BEN - COLBEN
Total number/ comparison		183	104	115	240	0	85
Cluster 1	136	23	25	8	73	0	10
Cluster 2	197	103	15	79	101	0	66
Cluster 3	96	32	40	25	10	0	5
